# Biomimetic Daytime Radiative Cooling Technology: Prospects and Challenges for Practical Application

**DOI:** 10.3390/ma18194556

**Published:** 2025-09-30

**Authors:** Jiale Wang, Haiyang Chen, Xiaxiao Tian, Dongxiao Hu, Yufan Liu, Jiayue Li, Ke Zhang, Hongliang Huang, Jie Yan, Bin Li

**Affiliations:** 1 School of Civil and Engineering, Hebei University of Architecture, Zhangjiakou 075000, China; 19556374484@163.com (J.W.); 13102639550@163.com (Y.L.); 18332548858@163.com (J.L.); 15832311239@163.com (K.Z.); huanghong.5055@163.com (H.H.); yanjie6253@126.com (J.Y.); 2 Hebei Key Laboratory of Diagnosis, Reconstruction and Anti-Disaster of Civil Engineering, Zhangjiakou 075000, China; 3 Hebei Collaborative Innovation Center of Green Buildings, Zhangjiakou 075000, China; 4 Hebei Tianhe Engineering Consulting Co., Ltd., Zhangjiakou 075000, China; 18730318985@163.com; 5 School of Materials Science and Engineering, University of Science and Technology Beijing, Beijing 100083, China; quanquanquan686@163.com

**Keywords:** bio-inspired radiative cooling, biological structures, photothermal regulation, refrigerating materials, multifunctionality

## Abstract

Biomimetic structures inspired by evolutionary optimized biological systems offer promising solutions to overcome current limitations in passive daytime radiative cooling (PDRC) technology, which efficiently scatters solar radiation through atmospheric windows and radiates surface heat into space without additional energy consumption. While structural biomimicry provides excellent optical performance and feasibility, its complex manufacturing and high costs limit scalability due to micro–nano fabrication constraints. Material-based biomimicry, utilizing environmentally friendly and abundant raw materials, offers greater scalability but requires improvements in mechanical durability. Adaptive biomimicry enables intelligent regulation with high responsiveness but faces challenges in system complexity, stability, and large-scale integration. These biologically derived strategies provide valuable insights for advancing radiative cooling devices. This review systematically summarizes recent progress, elucidates mechanisms of key biological structures for photothermal regulation, and explores their application potential across various fields. It also discusses current challenges and future research directions, aiming to promote deeper investigation and breakthroughs in biomimetic radiative cooling technologies.

## 1. Introduction

With the rapid industrialization, population growth, and climate warming worldwide, the demand for cooling has been constantly increasing, particularly in certain tropical regions. However, conventional compression-based cooling systems, such as traditional air conditioning and refrigeration units, not only require substantial electrical energy for operation but also predominantly utilize refrigerants with high global warming potential, thereby significantly exacerbating greenhouse gas emissions. This energy-intensive process results in excessive CO_2_ emissions and ozone depletion, leading to an energy crisis and environmental degradation [1,2,3]. It is estimated that cooling accounts for 15% of global electricity consumption and contributes to 10% of greenhouse gas emissions [4,5,6]. In this context, the transition from active cooling methods to passive cooling strategies that do not rely on external energy inputs could substantially reduce CO_2_ emissions. Consequently, the development of efficient and environmentally friendly cooling technologies to lower energy costs, mitigate greenhouse gas emissions, and address related thermal challenges has become an urgent scientific and technological priority.

**Figure 1:**
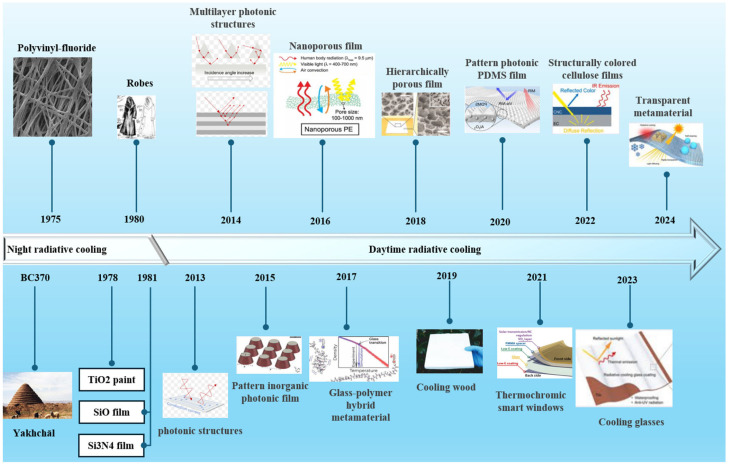
The timeline of PDRC development [26,74,75,76,77,78,79,80,81].

Radiative cooling, as an environmentally friendly passive cooling strategy, is marked by zero energy consumption and zero pollution [7,8]. Leveraging the substantial temperature gradient between the Earth (approximately 300 K) and outer space (around 2.7 K), excess heat can be dissipated via thermal radiation into the cosmos, enabling ubiquitous radiative cooling [9,10,11,12]. Due to its passive and renewable nature, significant advancements have been made in fields such as energy-efficient building design and personal thermal management [13]. In recent years, technological breakthroughs enabling daytime cooling below ambient temperatures have propelled radiative cooling into new research frontiers and broad practical applications (Figure 1), heralding a new chapter in cooling technology development [14,15,16,17,18,19,20,21,22,23,24].

Current research on radiative cooling, both domestically and internationally, primarily focuses on the design of novel radiative cooling materials and structures. To meet the evolving demands of future building environments, requirements for radiative cooling materials have become more stringent [25]. Several advanced materials have been developed that not only achieve effective radiative cooling effects but also exhibit excellent surface self-cleaning capabilities [26]. However, early traditional radiative cooling materials, such as white coatings and reflective films, face limitations in cooling efficiency and adaptability to diverse environmental conditions [27]. These materials generally possess low emissivity and insufficient solar reflectance, which restricts their overall cooling performance [28]. Table 1 summarizes the advantages and disadvantages of fundamental radiative cooling materials [14,29,30,31,32,33,34,35,36,37,38,39,40,41,42,43,44,45,46,47,48,49,50,51,52,53,54,55,56,57,58,59,60,61,62]. Although extensively studied, modern radiative cooling materials—such as silicon-based compounds, metal sulfides, polymers, antiparticle materials, and their composite coatings—exhibit optimized spectral performance within specific wavelength ranges, including high optical transmittance or reflectance, chemical inertness, and mechanical robustness. However, they often encounter inherent optical limitations, particularly the trade-off between transparency and high emissivity or reflectivity, as well as difficulties in maintaining thermal stability at high temperatures. Furthermore, manufacturing challenges like complex molding processes and environmental concerns, such as toxicity and long-term durability, present significant barriers to practical implementation.

**Figure 2:**
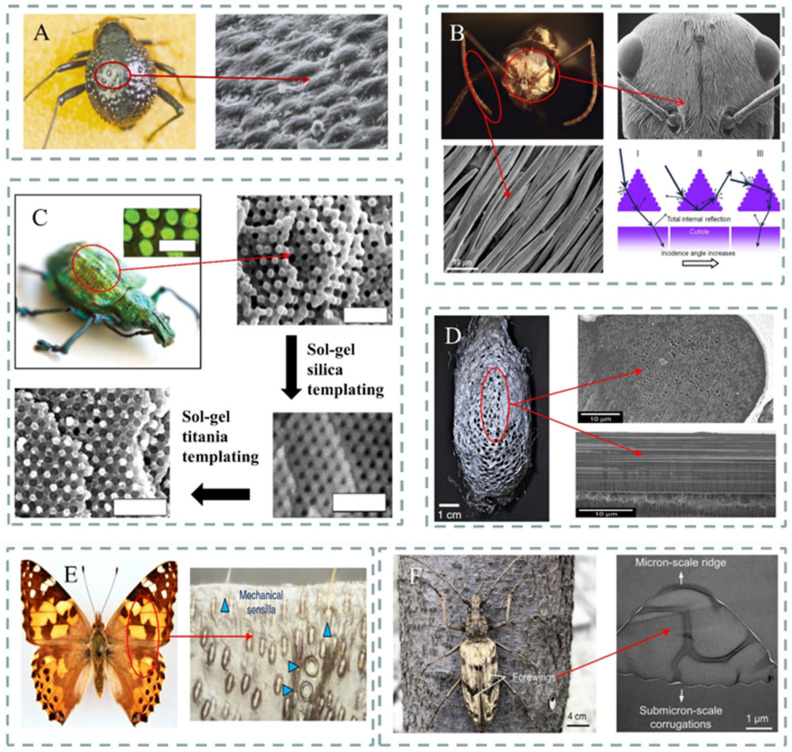
The developmental history of biomimetic radiative cooling: (**A**) scanning electron microscope images of *Stenocara* sp. and its elytra [68]; (**B**) photographs of Sahara silver ants, their head electron SEM images, and schematic diagrams illustrating their interaction with reflected sunlight [69]; (**C**) photographs of the proboscis beetle *L. augustus* [70] and optical images of its green photogenic scales; (**D**) scanning electron micrographs of the transverse and longitudinal sections of silkworms and silk fibers [90]; (**E**) images of butterflies and their micro-scale dendritic structures [73]; (**F**) photograph of *N. gigas* and transmission electron microscopy images of cross-sectional views of the setae [75].

**Figure 3:**
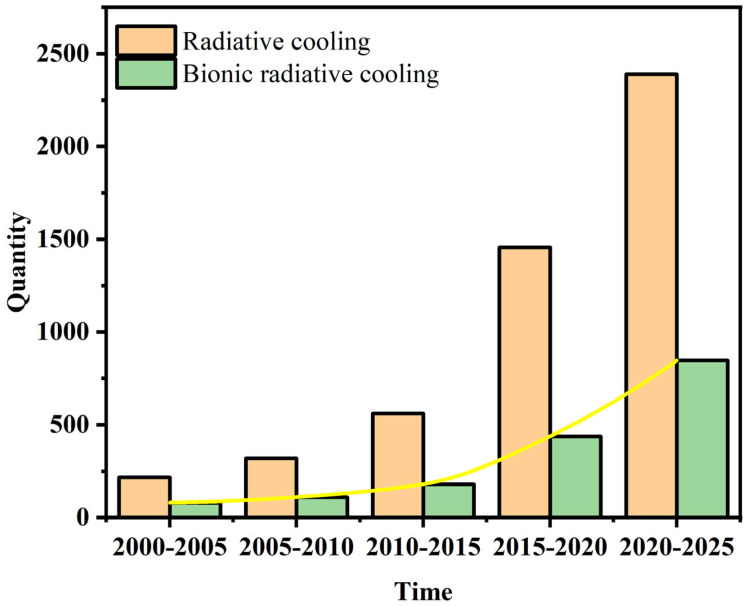
A comparative analysis of the publication trends in radiative cooling and biomimetic radiative cooling over recent decades. The yellow line represents the publication trend of this type of paper.

Therefore, effective PDRC requires materials with sufficiently high solar reflectance and mid-infrared (MIR) emissivity [63,64,65,66,67]. Through extensive evolutionary processes driven by natural selection, researchers have documented the development of specialized morphological features, such as the dorsal elytra of Coleoptera, characterized by a stochastic distribution of convex microstructures optimized for hygroscopic water collection from diurnal fog; the head, dorsal, and lateral surfaces of silver ants are covered with triangular cross-section setae, which induce total internal reflection at the interface between the setae and the air layer, enabling highly efficient reflection of solar radiation to survive in hot desert environments; the dual-scale setae of long-horned beetles serve two thermoregulatory functions—effectively reflecting sunlight and emitting thermal radiation—thus reducing body temperature [68,69,70,71,72,73] (Figure 2). These biological systems exhibit exceptional broadband solar reflectance and MIR emissivity, allowing them to adapt to complex and harsh environments, providing invaluable inspiration for advanced PDRC design. Figure 3 illustrates publication trends of radiative cooling literature over recent decades, with biomimetic radiative cooling studies showing a rapid and consistent increase in annual publications, particularly in recent years, indicating that the development of bio-inspired radiative cooling has become more mature. To date, drawing inspiration from nature turns out to be a viable approach for addressing the challenges faced by conventional technologies.

Recently, numerous reviews have been issued primarily focusing on bio-photonics, material interactions, and biomimetic optical materials; however, their core emphasis does not center on radiative cooling [82,83,84,85]. Existing reviews on radiative cooling predominantly address fabrication techniques, material properties, and applications [86,87,88,89], thereby largely neglecting a critical aspect: the design strategies of radiative coolers, particularly biomimetic design approaches. This oversight hampers the advancement of biomimetic radiative cooling technologies. Consequently, it is essential to conduct a timely and comprehensive review of developments in this field to facilitate ongoing technological progress. This paper starts with an overview of the fundamental principles and design guidelines of PDRC. It then discusses various biomimetic-inspired design and research efforts. Subsequently, the potential applications of biomimetic PDRC technologies in building cooling, electronic device thermal management, and personal heat regulation are briefly summarized. Finally, a comprehensive and objective outlook on future directions is offered.

## 2. Fundamental Principles of Radiative Cooling

**Figure 4:**
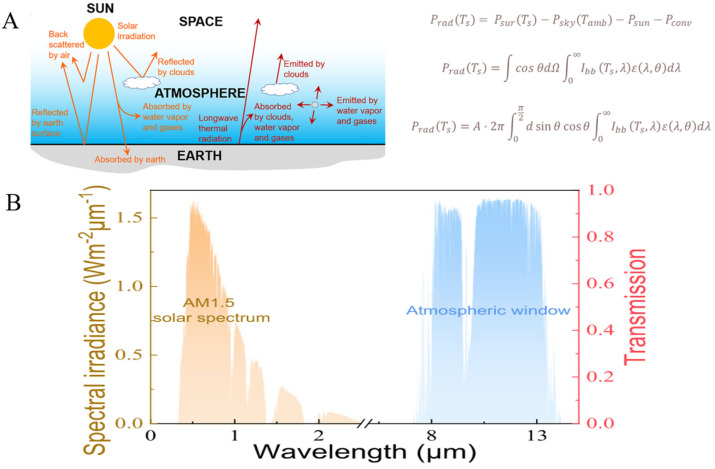
(**A**) Schematic diagram of the heat exchange process in passive daytime radiative cooling [92]; (**B**) spectral profiles of solar radiation and atmospheric transmittance [94].

All objects with temperatures above 0 K continuously absorb and emit electromagnetic radiation. Through this process of absorption and emission, energy exchange is held between objects at different temperatures [91]. Heat from the Earth (approximately 300 K) can be radiatively transferred to outer space (around 2.7 K) via the atmospheric window in the 8–13 μm wavelength range, facilitating cooling, particularly during nighttime when solar radiation is not present [92,93]. During the daytime, if the solar irradiance incident on a radiative cooling device is minimal, it can achieve PDRC in addition to radiative heat emission (Figure 4A).

In practical implementations, the thermal regulation efficacy of PDRC systems is governed not solely by solar absorptance and thermal emissivity but also by atmospheric radiative exchange processes and non-radiative heat transfer mechanisms such as convection and conduction. Atmospheric radiative transfer involves emission from gas molecules and aerosol particles within the atmosphere, while non-radiative heat transfer primarily depends on conductive and convective processes surrounding the radiative cooler.

Therefore, the net cooling capacity of the PDRC can be expressed as follows [94]:

(1) $$P_{rad}\left(T_{s}\right)=\;P_{sur}\left(T_{s}\right)-P_{sky}\left(T_{amb}\right)-P_{sun}-P_{conv}$$

Here, $P_{rad}(T_{s})$ denotes the net radiative cooling power at the $T_{s}\;$ surface temperature; $P_{sur}\left(T_{s}\right)$ represents the radiative power at $T_{s}\;$ surface temperature $\;P_{sky}(T_{amb})$; $P_{sun}$ indicates the incident solar energy absorbed by the surface during daytime; and $\;P_{conv}$ signifies convective heat transfer. Therefore, to achieve effective net radiative cooling, $P_{rad}\;$ should be maximized while $P_{sky}$, $P_{sun}and$ $P_{conv}$ are minimized.

The radiative power emitted from the surface of the radiative cooler (*P_rad_*) is a function of the surface temperature and the emission spectrum and can be expressed as follows:

(2) $$P_{rad}\left(T_{s}\right)=\int \cos\theta d\Omega \int_{0}^{\infty }I_{bb}\left(T_{s},\lambda \right)\varepsilon \left(\lambda ,\theta \right)d\lambda$$

where *Ω* represents the solid angle; *θ* is the angle between the surface normal and the direction of the solid angle; $T_{s}$ denotes the absolute temperature of the radiative cooler surface; $\varepsilon \left(\lambda ,\theta \right)$ is the surface emissivity, which varies with surface angle and wavelength; $I_{bb}$ $\left(T_{s},\lambda \right)$ is the spectral radiance of a blackbody at temperature $T_{s}$; and the integral over the hemisphere, $\int d\Omega =\int_{0}^{\frac{\pi }{2}}\sin\theta d\theta$ accounts for the angular distribution. Consequently, the radiative power, $P_{rad}\left(T_{s}\right)$ can also be expressed as follows [94]:

(3) $$P_{rad}\left(T_{s}\right)=A\cdot 2\pi \int_{0}^{\frac{\pi }{2}}d\sin\theta \cos\theta \int_{0}^{\infty }I_{bb}\left(T_{s},\lambda \right)\varepsilon \left(\lambda ,\theta \right)d\lambda$$

where *A* represents the surface area of the radiative cooler. Furthermore, according to Planck’s law, the thermal radiation emitted by an object depends on the fourth power of its surface temperature. $I_{bb}$ $\left(T_{s},\lambda \right)$ can be expressed as follows [94]:

(4) $$I_{bb}\;\left(T_{s},\lambda \right)=\frac{2hc^{2}}{\lambda^{5}\left(e^{\frac{hc}{\lambda \kappa T_{s}}}-1\right)}$$

Here, *h* is the Planck constant; *k* is the Boltzmann constant; *c* is the speed of light. Enhancing the thermal radiation of the radiative cooler’s surface can improve its net cooling efficiency.

The solar reflectance, denoted as $\bar{\gamma }$, is derived from the material’s spectral reflectance and the spectral solar irradiance, *I_solar_*.

(5) $$\bar{\gamma }=\frac{\int_{0.3\mu m}^{2.5\mu m}I_{solar}\left(\lambda \right)\gamma \left(\lambda \right)d\lambda }{\int_{0.3\mu m}^{2.5\mu m}I_{solar}\left(\lambda \right)d\lambda }$$

The effective thermal emissivity, $\bar{\varepsilon }$, is calculated based on spectral emissivity and the cooling potential, which is the difference between the emitted energy, $I_{b}$, and the spectral atmospheric energy, $I_{atm}$.

(6) $$\bar{\varepsilon }=\frac{\int_{4\mu m}^{20\mu m}{\;[I}_{b}(\lambda )-I_{atm}(\lambda )]\varepsilon (\lambda )d\lambda }{\int_{4\mu m}^{20\mu m}{\;[I}_{b}(\lambda )-I_{atm}(\lambda )]d\lambda }$$

The formula for calculating cooling power is as follows:

(7) $$q_{coolimg}=q_{rad}-q_{atm}-q_{solar}-q_{loss}$$

Here, $q_{rad}$ denotes the radiative power of the material, expressed as follows:

(8) $$q_{rad}=2\pi \int_{0}^{\infty }\int_{0}^{\frac{\pi }{2}}\varepsilon \left(\lambda ,\theta \right)I_{b}\left(\lambda ,T\right)\sin\theta \cos\theta d\theta d\lambda$$

where *ε* represents emissivity, *λ* denotes wavelength, *θ* is the latitude angle, and $I_{b}\;$ is the spectral radiance of a blackbody. The formula for the radiative flux absorbed by the atmosphere, $q_{atm}$, is given by

(9) $$q_{atm}=2\pi \int_{0}^{\infty }\int_{0}^{\frac{\pi }{2}}\alpha \left(\lambda ,\theta \right)I_{atm}\left(\lambda ,T_{a}\right)\sin\theta \cos\theta d\theta d\lambda$$

where *α* represents the absorption coefficient, $I_{atm}$ denotes the spectral radiance of the atmosphere, which can be modeled using the MODTRAN 5 software [74]. In Equation (7), $q_{solar}$ signifies the absorbed solar energy, calculated as follows:

(10) $$q_{solar}=\int_{0}^{\infty }I_{solar}\left(\lambda \right)\alpha \left(\lambda \right)d\lambda$$

The term $q_{loss}$ in Equation (7) represents the intrinsic cooling loss of the material. Since our simulation assumes that the material temperature equals the ambient air temperature, this term can be neglected [95].

According to the energy balance equation, the characteristics of an ideal PDRC system can be summarized into three key points. First, it should exhibit strong solar reflectance to minimize solar irradiance ($P_{sun}$). Second, it must possess high thermal emissivity to maximize radiative heat loss ($P_{rad}$) while concurrently minimizing sky radiance ($P_{sky}$). Third, it should demonstrate significant insulation properties to reduce non-radiative heat transfer, particularly convection ($P_{conv}$) (Figure 4B). Consequently, the PDRC must ensure highly selective emission within the transparent atmospheric window while minimizing the effects of convective and conductive heat transfer. In most experimental setups, radiative coolers are insulated with polystyrene foam and enclosed with abnormal infrared transmittance polyethylene (PE) films to suppress heat gain from the surrounding environment [96]. Taking into account this design principle, various cooling systems have been developed [97,98,99,100]. Therefore, when designing radiative coolers, it is critical to consider the relevant application scenarios to accurately evaluate their cooling performance without overestimating or underestimating their capabilities.

## 3. Biomimetic Passive Daytime Radiative Cooling

Founded on the principles of PDRC, achieving sub-ambient cooling requires that radiative coolers emit thermal radiation within the MIR spectrum. Crucially, they must also exhibit high broadband reflectivity of solar radiation, coupled with elevated infrared emissivity to mitigate parasitic heat gain from solar absorption. Historically, the simultaneous realization of high solar reflectance and strong infrared emissivity has posed significant challenges, resulting from the complexity of micro- and nano-structural design and the selection of suitable materials. Advances in biological observation and research have unveiled various biological structures and mechanisms that enhance broadband reflectivity and infrared emission, offering valuable insights for material design. Concurrently, developments in material synthesis and fabrication techniques have resulted in the creation of numerous bio-inspired daytime radiative cooling devices [101,102,103,104,105,106,107,108,109,110,111], thereby fostering the rapid growth of PDRC technology. This section systematically reviews bio-structural and bio-material mimetics, as well as a multitude of adaptive biomimetic materials guided by biological systems.

### 3.1. Structural Biomimicry

Organisms must balance the essential need for sunlight with its detrimental effects, such as heat stress and increased predation risk. In response, numerous species—including desert silver ants, beetles, longhorn beetles, and cicadas—have evolved sophisticated structural solar reflectors. These structures are indispensable adaptations that enable effective camouflage, facilitate signal communication, and maintain optimal body temperatures, thereby supporting key physiological functions [73,75,90,112,113,114,115,116,117,118,119,120,121,122,123,124,125,126,127,128]. Based on their structural features and functional mechanisms, these adaptations can be categorized into three distinct groups for detailed discussion.

#### 3.1.1. Surface Micro- and Nanostructures

**Figure 5:**
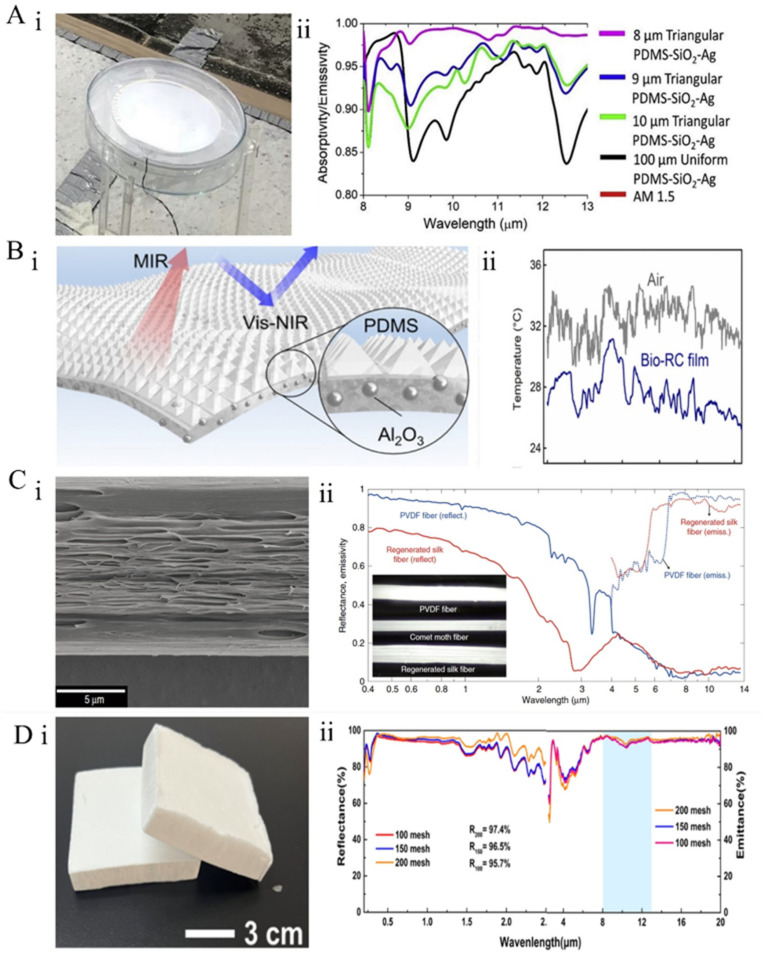
Micro- and nanostructured biomimetic architectures: (**A**) Sahara silver ant mimicry [101]: (**i**) schematic of a PDMS-SiO_2_-Ag daytime passive radiative cooler; (**ii**) emissivity of patterned PDMS-SiO_2_-Ag with a trihedral array. (**B**) Tropical longhorn beetle *Neocerambyx gigas* mimicry [75]: (**i**) schematic of a bio-inspired flexible hybrid film; (**ii**) time-dependent temperature data of air and Bio-RC film under direct sunlight. (**C**) Mimicry of the *Argema mittrei* silkworm cocoon [90]: (**i**) SEM image of longitudinal cross-section of PVDF fibers containing high-density voids; (**ii**) combined hemispherical reflectance and emissivity spectra of a ~100 μm thick regenerated silk fiber bundle and a ~100 μm diameter PVDF fiber. (**D**) Butterfly mimicry [114]: (**i**) optical image of CAG; (**ii**) solar reflectance and infrared emissivity spectra of various super-surface CAGs.

In nature, certain organisms have evolved specialized thermal dissipation structures that work sympathetically with their tissues and organs to regulate body temperature through millions of years of natural selection and evolution [75,112]. For instance, the Sahara silver ant [112] can survive in temperatures up to 70 °C, thanks to its covering of silvery hair, which exhibits a distinctive triangular cross-section composed of two wavy top facets and a flat bottom facet closely adhering to the body surface (Figure 2B). Guided by this, Jeong and colleagues [101] developed a PDMS thin film modified with a temperature-regulating prismatic structure (Figure 5A(i)). This film achieves an average emissivity of 0.98 within the 8–13 μm atmospheric transparency window, significantly surpassing the 0.92 of uniform planar polydimethylsiloxane (PDMS). This enhancement is assigned to the gradient refractive index effect introduced by the triangular prism array, which amplifies Mie scattering and thermal radiation efficiency. (Finite-Difference Time-Domain) FDTD simulations suggest that a triangular structure with an 8 μm feature size can increase emissivity by 6.5%, approaching near-ideal cooling performance (Figure 5A(ii)).

Similarly, the tropical longhorn beetle (*Neocerambyx gigas*) native to Southeast Asia exhibits thermotolerance attributed to its unique micro–nanostructures on the surface of its forewings. These forewings are densely covered with over 25,500 fine hairs per square centimeter (Figure 2F), which naturally constitute a finely structured triangular cross-section. This structure serves dual thermoregulatory functions: effectively reflecting sunlight and emitting thermal radiation, thereby narrowing the beetle’s body temperature. Inspired by this discovery, researchers [75] fabricated a photonic film composed of a micro-pyramid array polymer matrix integrated with randomly distributed ceramic particles (Figure 5B(i)). The film reflects approximately 95% of solar irradiance and exhibits an infrared emissivity exceeding 0.96. Its effective cooling power reaches approximately 90.8 W·m^−2^. The Bio-RC membrane was exposed to direct sunlight under clear sky conditions, with ambient air and membrane temperatures recorded during the peak heat period of the day, with recorded temperature drops of up to 5.1 °C under direct sunlight (Figure 5B(ii)). The micro–nanostructures found in Sahara silver ants and longhorn beetles sympathetically enhance solar reflectance and infrared emission, facilitating exceptional thermoregulation.

Similar structures have likewise been observed within silkworm cocoons. The cocoon fibers of the comet moth *Argema mittrei* contain high-density air micropores, which effectively protect the pupae inside from overheating when exposed to direct sunlight (Figure 2C). Single silk fibers with a diameter of 50 μm can reflect up to 66% of incident sunlight and exhibit a high mid-infrared emissivity of 0.88, enabling the cocoon to act as an efficient PDRC device.

Inspired by natural fibers, Norman et al. [90] developed biomimetic nanostructured fibers based on regenerated silk fibroin and polyvinylidene fluoride (PVDF) through wet spinning (Figure 5C(i)). Optical characterization indicates that these fibers demonstrate exceptional optical properties for radiative cooling applications: the nanostructured regenerated silk fibers achieve a solar reflectance of 0.73 and a thermal emissivity of 0.90, while the nanostructured PVDF fibers attain a solar reflectance of 0.93 and a thermal emissivity of 0.91 (Figure 5C(ii)). The filamentous voids induce high directional scattering, imparting the fibers with a high reflective gloss.

It is a known fact that micro- and nanoscale pores within transparent matrices can enhance reflection through scattering, while in matrices with inherent absorption, scattering caused by internal pores amplifies the absorption process. The dorsal forewing surface of butterflies (Figure 2E) is less covered by four types of wing scales; Tsai et al. [73] observed that different scales exhibit distinct emissivities, with scent patch scales displaying nearly uniform and exceptional emissivity. Notably, the outstanding emissivity of scent patches is attributed to their unique microstructure, which is full of a randomly porous chitin fiber network. This disordered porous architecture maximizes MIR scattering, with each scattering event promoting absorption of MIR radiation by adjacent chitin fibers, resulting in the strongest luminescence among all wing scale types [113]. Guided by this structure, Cai et al. [114] developed a sustainable cellulose nanocrystal aerogel (CAG) grating (Figure 5D(i)), featuring a tunable super-surface capable of high-efficiency radiative cooling. Simulations showed that the regular channels and irregular grooves on the aerogel surface function as diffraction gratings, facilitating the diffraction of sunlight and broadband reflection. This CAG exhibits an ultra-high solar reflectance of 97.4% and a high infrared emissivity of 94% (Figure 5D(ii)). This work provides valuable insights into the design of biomimetic thermoregulatory materials for energy conservation.

#### 3.1.2. Photonic Crystal Structure

Photonic crystals are artificially engineered microstructures composed of media with varying refractive indices arranged periodically in space. Their fundamental characteristic is the formation of photonic band gaps, which enable selective blocking of light propagation at specific frequencies while allowing other frequencies to transmit with high efficiency. Nature provides exemplary models of such structures; for instance, the natural multilayered fish scales [115,116] where guanine crystals (high refractive index medium, n ≈ 1.83) alternate periodically with cytoplasm (low refractive index medium), forming a one-dimensional photonic crystal. The Bragg scattering effect within this structure produces broadband high reflectivity independent of wavelength in the visible spectrum, conferring a bright silvery appearance to the scales. This structure achieves high reflectivity through multilayer interference mechanisms, permitting fish to dynamically match environmental lighting conditions—such as tail light spectra-for effective camouflage.

**Figure 6:**
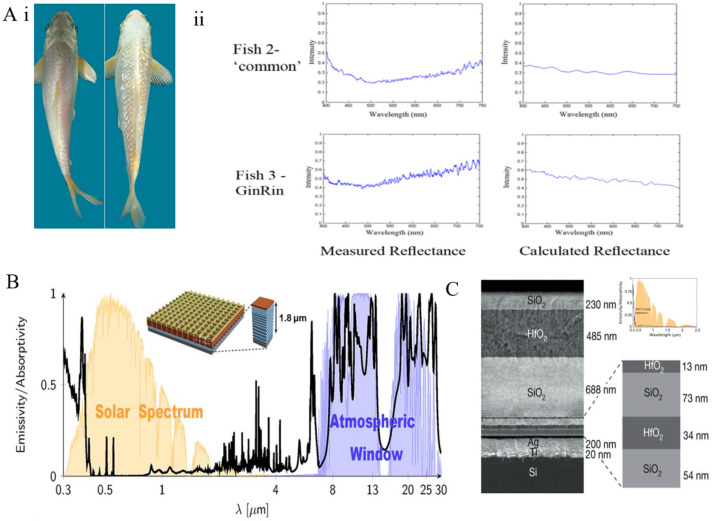
Photonic crystal structures [115]: (**A**) Koi fish: (**i**) photographs of standard Koi and Gin Rin Koi; (**ii**) normalized measured reflectance, and simulated reflectance. (**B**) Emissivity of multilayer structures composed of photonic crystal layers [117]. (**C**) SEM image of a photonic radiative cooler constructed from seven layers of HfO_2_ and SiO_2_ [64].

Taking *Gin Rin* koi as an example (Figure 6A), the number of iridophore cells within the scales increases, and the stacking layers of crystals and cytoplasm within each cell are significantly augmented. Additionally, the crystal orientation is optimized to form a staggered arrangement, leading to reduced stacking periodicity and increased disorder in interlayer spacing. These structural modifications enhance the Bragg scattering strength of the photonic crystal and broaden the photonic band gap, ultimately increasing the reflectance to over 1.5 times that of standard koi, providing critical insights for biomimetic broadband reflectors. Researchers have designed artificial photonic crystal thin films by precisely engineering multilayer structures that exploit the periodic stacking of high- and low-refractive-index materials to induce interference effects and phonon-polariton resonances. In preliminary work by the Fan team [117], biomimetic multilayer structures on silver substrates with MgF_2_/TiO_2_ reflective layers coupled with SiC/α-quartz infrared emitters achieved a solar spectrum reflectance of 96.5% and high emissivity within the atmospheric window (ATW). The photonic band structure was meticulously tailored to meet the spectral requirements of solar reflection and thermal radiation, resulting in a net cooling power exceeding 100 W/m^2^ under ambient conditions (Figure 6B).

Subsequent optimization of a seven-layer alternating HfO_2_/SiO_2_ photonic crystal structure [64] (Figure 6C) involved controlling the interference effects of sub-100-nanometer layers to enhance short-wavelength reflection, while the phonon vibrational properties of the top sub-micrometer layers amplified mid-infrared emission. This design achieved a solar reflectance of 97% and significant emission within the atmospheric window, with experimental measurements detecting a sub-environment temperature reduction of 4.9 °C. Although the HfO_2_-SiO_2_ photonic radiative cooler demonstrated substantial temperature drops below ambient, its MIR emissivity and reflectance in the shortwave visible and ultraviolet regions remain areas for further improvement. To address these challenges, researchers have explored material system selection and structural optimization, developing multilayer photonic coolers such as TiO_2_-SiO_2_ [118], Al_2_O_3_-SiO_2_ [119], and Si_3_N_4_-SiO_2_ [120], which exhibit superior optical properties and cooling performance compared to HfO_2_-SiO_2_.

#### 3.1.3. Hierarchically Porous Structure

Photonic-structure-based PDRC devices typically require metallic layers to account for sunlight. However, this not only complicates manufacturing and increases costs but also restricts applicability in fields such as architecture. Theoretical simulations suggest that pore features on the order of tens of nanometers can scatter ultraviolet radiation, while pore sizes of several hundred nanometers can scatter visible light [65]. When pore diameters are extended to several micrometers, they exhibit strong mid-infrared scattering. For layered structures, when pore sizes or pore dimensions approach the target wavelength, their operational mechanism primarily relies on Mie scattering. Consequently, PDRC devices with layered architectures can effectively scatter solar radiation [121].

**Figure 7:**
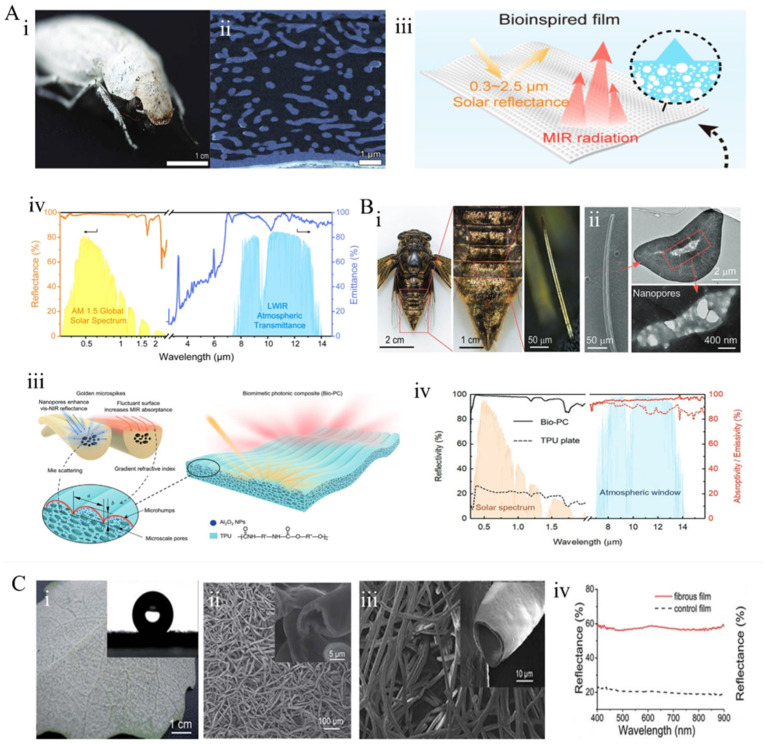
Layered porous structures: (**A**) Bright white beetle *Cyphochilus* sp. [122]: (**i**) macro-photograph and optical microscopy image showing the scale arrangement on the elytra; (**ii**) high-resolution computed tomography (CT) scan slices; (**iii**) schematic diagram of the biomimetic membrane [125]; (**iv**) spectral solar reflectance and mid-infrared (MIR) emissivity of the biomimetic membrane. (**B**) (**i**) Photograph of *Cryptotympana atrata* cicada [108] and (**ii**) microscopic image of a single golden micro-spike, along with SEM, cross-sectional SEM, and TEM images of the golden micro-spike; (**iii**) Multiform composite radiative cooling membrane composed of porous micro-spike structures; (**iv**) solar spectral reflectance and MIR spectral emissivity of the biomimetic thin film. (**C**) *Poplar* leaf [127]: (**i**) digital photograph of the abaxial surface; (**ii**) SEM image of the banded trichomes on the leaf underside; (**iii**) SEM image of the fibrous membrane; (**iv**) reflectance spectra of the fibrous membrane (solid line) compared to a control membrane without fibers (dashed line).

In nature, various organisms have evolved intricate, layered porous structures to harness Mie scattering for efficient broadband solar reflection, with beetles being used as prominent examples. For instance, the *Cyphochilus* spp. beetles found in equatorial regions exhibit remarkable whiteness [68] (Figure 7A(i)). Their scales contain a disordered three-dimensional network formed within a mere 5-micrometer-thick chitinous layer, composed of interconnected nanofibrils approximately 250 nm in diameter. This high volume fraction, accounting for 70%, significantly enhances the density of scattering centers (Figure 7A(ii)), thereby enhancing scattering intensity. The anisotropic shape of the scatterers narrows the angular dependence of scattering, ensuring the scales appear white from any viewing angle [122]. This highly optimized layered porous network results in an exceptionally rapid transport mean free path—approximately 1.40 to 1.47 μm—one of the lowest recorded among biological materials, conferring extraordinary scattering efficiency within a minimal thickness [123,124]. He et al. [125] replicated the natural photonic structure of the *Cyphochilus* beetle’s wings to achieve high-efficiency PDRC. They employed scalable phase separation and rapid hot-pressing techniques to fabricate bio-inspired photonic films composed of a surface-ordered pyramid array with a base edge length of 4 μm, along with numerous internal nanovoids and micropores (Figure 7A(iii)). The optimized pore architecture and surface-enhanced photonic array yielded an average solar reflectance of approximately 98% and a high infrared emissivity of about 96% (Figure 7A(iv)).

Similar to this beetle, the cicada [108] (*Cryptotympana atrata*) employs a unique layered porous structure that facilitates a strong backscattering mechanism, effectively reflecting sunlight to reduce solar absorption (Figure 7B(i,ii)). Coupled with the high infrared emissivity characteristic of biological materials, this synergistically enables passive radiative cooling of the organism. Inspired by this mechanism, researchers have developed a microimprinting technique combined with phase separation methods to fabricate biomimetic photonic materials composed of porous polymer–ceramic composites molded into micro-cusp structures (Figure 7B(iii)). These composites exhibit high solar reflectance (97.6%) and infrared emissivity (95.5%) within the atmospheric window, resulting in a midday cooling power of 78 W·m^−2^ and a maximum sub-ambient temperature reduction of 6.6 °C (Figure 7B(iv)). Furthermore, this approach helps with the multiform fabrication of composite materials beyond thin films, such as additive manufacturing into conventional three-dimensional structures. This work serves as a biomimetic pathway for the development of high-performance thermal regulation materials and devices.

Certain plants, such as *Leontopodium nivale*, *Boehmeria nivea*, and *Poplar*, have evolved a white, hairy surface layer on their leaves. These white, hairy layers exhibit similar structural features, comprising a three-dimensional porous framework assembled from numerous micro- and nanoscale fibers in a random stacking arrangement [126,127,128]. The pores and fibers, with sizes comparable to solar wavelengths, are considered effective broadband scatterers that reflect sunlight, thereby reducing water loss brought about by high-temperature irradiation and preventing leaf desiccation. Notably, the hollow core–shell fiber structures on the surface of *Poplar* leaves (Figure 7C(i,ii)) demonstrate, as opposed to homogeneous solid fibers, a higher backscattering intensity and reflectance per unit thickness. Inspired by this, researchers employed coaxial electrospinning to precisely replicate the hollow fiber architecture of Poplar leaf trichomes, achieving a visible light reflectance of up to 60–70% (Figure 7C(iii)), significantly surpassing traditional flat coatings (approximately 20%), effectively scattering sunlight and reducing surface temperatures. Additionally, the membrane surface exhibits excellent superhydrophobicity (contact angle of 147.0 ± 1.1°, close to the natural trichome contact angle of 146°) (Figure 7C(iv)), which effectively resists water infiltration and erosion, thereby extending service life. Moreover, this technique is compatible with various conventional polymers, and by adjusting spinning parameters, it can be tailored to meet different application requirements. Overall, this approach can reduce air conditioning energy consumption by over 20%, providing material support for mitigating urban heat island effects and global carbon reduction efforts. These organisms’ unique structures serve as biomimetic templates for the design of highly efficient optical scatterers in radiative cooling materials.

### 3.2. Material Biomimicry

**Figure 8:**
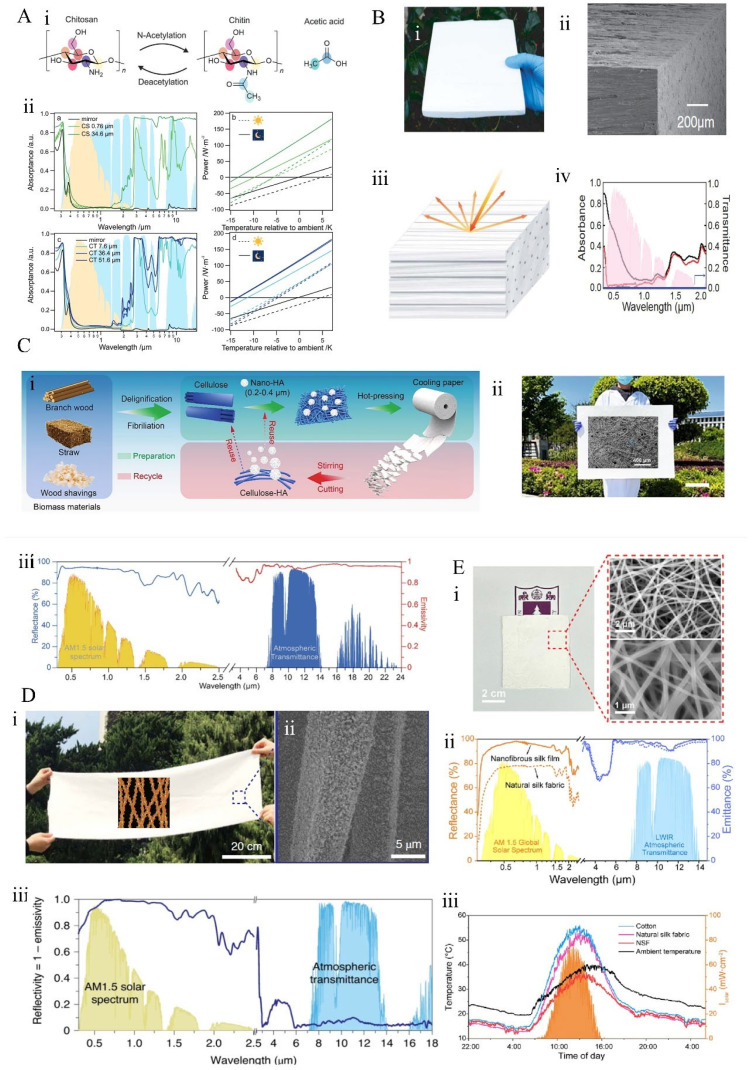
Biomimetic materials for radiative cooling: (**A**) Bio-inspired chitosan and shellac films for radiative cooling [129]: (**i**) chemical structures of chitosan, chitin, and acetic acid; (**ii**) spectral absorption rates of chitosan and chitin films of varying thicknesses on silver mirror substrates, along with the computed cooling capacities as functions of temperature below ambient conditions. (**B**) Cooling wood fabricated from natural timber [76]: (**i**) photograph and (**ii**) SEM images of the cooling wood; (**iii**) The reflective structure of the material; (**iv**) solar spectral absorptance of natural cooling wood. (**C**) Radiative cooling paper composed of delignified and fibrillated cellulose fibers combined with nanoscale hydroxyapatite via established pulping and papermaking processes [130]: (**i**) photograph and (**ii**) SEM images of the cooling paper; (**iii**) solar reflectance and infrared emissivity of the cooling paper. (**D**) Nano-engineered silk fibers for radiative cooling [134]: (**i**) schematic illustrating the attachment of Al_2_O_3_ nanoparticles to silk fibers, enhancing UV resistance; (**ii**) SEM image of the nano-engineered silk textile; (**iii**) spectral reflectance of the nano-engineered silk fabric. (**E**) Nanofiber fibroin membranes for radiative cooling [135]: (**i**) photograph and SEM images of the fibroin membrane; (**ii**) spectral reflectance and emissivity of the fibroin membrane; (**iii**) outdoor cooling performance of tested fibroin membranes.

Unlike biological structures that replicate geometric morphology, biological materials not only exhibit excellent radiative properties within the MIR wavelength band but also possess degradability. Further processing of biological materials to allow high solar reflectance can facilitate more environmentally friendly daytime radiative cooling. Lauster et al. [129] explored the potential of chitin and its N-deacetylated form, chitosan, in passive radiative cooling (Figure 8A(i)). By combining chitosan or chitin films with reflective backing materials, the composite films achieve a low solar absorption rate of 3.1–6.9%, depending on film thickness, along with a high MIR emissivity, resulting in a temperature drop of 3.5 °C below ambient conditions. Natural wood typically exhibits a strong MIR emissivity (Figure 8A(ii)), but due to lignin content, it has a penetrating solar absorption rate, rendering it unsuitable as a daytime radiative cooler. Li et al. [76] performed complete delignification and densification of natural wood to produce cooling wood composed of multi-scale cellulose nanofibers (Figure 8B(i,ii)). These cellulose nanofibers can backscatter sunlight (Figure 8B(iii)), inducing a 96% solar reflectance (Figure 8B(iv)). Similarly, Sun et al. [130] fabricated a recyclable, biodegradable cooling paper via mature pulping and papermaking processes, consisting of delignified and fibrillated cellulose fibers and nanoscale hydroxyapatite (Figure 8C(i,ii)). The resulting cooling paper demonstrated a solar reflectance of 94% and an MIR emissivity of 0.95 (Figure 8C(iii)), with complete degradation in soil within four months. Moreover, sustainable and environmentally friendly cellulose derived from wood can be extracted and subsequently assembled [131], phase-separated [132], electrospun [133], or processed through other methods to produce thin films or coatings for PDRC applications.

Silk is a naturally occurring radiative cooling material, distinguished by its proteinaceous composition, which exhibits high absorption within the ultraviolet spectrum. Consequently, its overall solar reflectance is restricted to approximately 86%, thereby preventing daytime cooling below ambient temperatures. To enhance ultraviolet reflectance without compromising the intrinsic hierarchical structure and unique constituents of silk, Zhu et al. [134] employed a scalable silane coupling agent-assisted dip-coating technique to affix Al_2_O_3_ nanoparticles onto the silk fibers (Figure 8D(i,ii)). This modification increased the ultraviolet reflectance of silk from 70% to 85%, with concomitant improvements in reflectance within the visible-near infrared (VIS-NIR) range, resulting in a total solar spectrum reflectance of 95% (Figure 8D(iii)). Leveraging silk’s inherent high emissivity in the MIR domain, the nanostructured silk achieved a daytime temperature reduction of 3.5 °C below ambient. In contrast, He et al. [135] extracted fibroin from silkworm cocoons and fabricated nanofiber fibroin membranes via electrospinning (Figure 8E(i)). These nanofiber membranes preserved the high MIR emissivity characteristic of pristine silk and enhanced solar reflectance through their fibrous architecture (Figure 8E(ii)), resulting in superior daytime cooling performance (Figure 8E(iii)).

In summary, the reconstruction of biological structures using synthetic materials to achieve high solar reflectance and infrared emissivity, or the secondary processing of certain biological materials to further enhance their multi-band optical properties, has been demonstrated as an effective strategy for improving the performance of radiative coolers. This approach offers valuable insights for the future development and application of radiative cooling technologies, particularly in the realm of biomimetic radiative cooling systems [136,137].

### 3.3. Adaptive Bionics

Efficient PDRC systems are desirable during the hot summer months; however, they are less suitable in transitional seasons and cold winters, as a solely cooling function can result in excessive cooling capacity, increased thermal loads, and energy wastage [138]. Consequently, smart radiative coolers (SRCs) equipped with environmentally adaptive thermal regulation capabilities have garnered significant attention due to their promising application prospects [94,139,140]. Essentially, materials or structures exhibiting environment-dependent spectral and thermal modulation responses can be established as potential SRCs. In ecosystems, numerous organisms demonstrate remarkable environmental spectral modulation for functions such as body temperature regulation, camouflage, signal transmission, and intra- and inter-species communication [141], with chameleons [142] and cephalopods [143,144] serving as prominent examples. These environment-responsive spectral modulation mechanisms offer valuable insights for the design and development of SRCs. This section reviews biological mechanisms of environment-adaptive spectral modulation and explores their biomimetic applications in SRC technology.

#### 3.3.1. Thermal-Induced Adaptive Response

**Figure 9:**
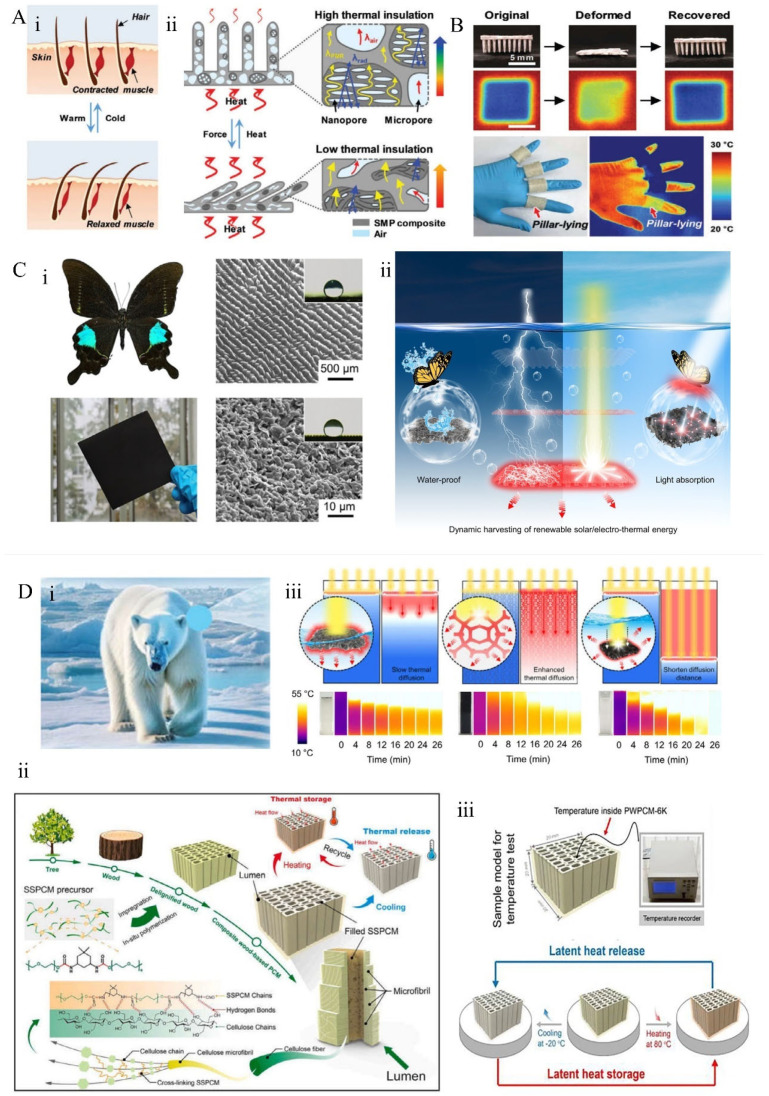
Biological thermally induced adaptive responses: (**A**) thermally induced adaptive mechanisms of human skin [146] and hair structures under varying thermal environments [147]; (**i**) morphological deformation mechanisms of hairy SMPs; (**ii**) A shape memory polymer with a layered micro-nanopore structure of hair texture that can switch between a straight/open state and a curled/closed state in response to temperature stimuli, thereby dynamically adjusting thermal insulation.; (**B**) the original, deformed, and recovered states of hairy SMPs [148], along with corresponding infrared thermography measurements; (**C**) (**i**) photographs and SEM images of *Papilio paris Linnaeus* butterfly wings and SETC [149]; (**ii**) photographs of PW during electrothermal charging processes and (**iii**) infrared images showing temporal temperature variations; (**D**) (**i**) photograph of the polar bear; (**ii**) preparation process and microstructural schematic of PWPCM [150]; (**iii**) experimental design for the temperature evolution of composite PCM.

In nature, the fur and feathers of birds and mammals play a key role in thermoregulation [145]. These structures possess porous characteristics that effectively reflect infrared radiation and are adapted to environmental changes for efficient heat management. Human hair [146] also demonstrates significant thermoregulatory capabilities (Figure 9A(i)); when external temperatures decrease, the arrector pili muscles at the hair follicle base contract, causing hair to stand upright. This upright position improves the air layer on the skin surface, enhancing insulation and reducing heat loss. Shape memory polymers (SMPs) can respond to temperature variations by changing their molecular structures and phase transition properties, exhibiting biomimetic thermal control behaviors. Choe et al. [147] developed a hair-patterned SMP (Figure 9A(ii)) with a layered micro–nanoporous structure, capable of switching between straight/open and curled/closed states in response to temperature stimuli, thereby dynamically modulating thermal insulation. Experimental results indicated a significant increase in insulation performance in the straight state, with a temperature difference (ΔT) reaching 17.16 °C, while the layered state reduced ΔT to 6.62 °C. The switching ratio of insulation performance was 2.59, demonstrating excellent dynamic thermal regulation. In another study, Wang et al. [148] drew inspiration from the adaptive behavior of Mimosa pudica to develop a thermally activated SMP composed of polycaprolactone (PCL) and polycaprolactone elastomer (PCLE) (Figure 9B). The PCLE material exhibited outstanding shape fixation, recovery capabilities, and high repeatability of shape memory. By integrating a specially designed deformable bilayer structure as an intermediate layer, they successfully created an intelligent protective garment with enhanced impact resistance and thermal insulation. After heating at 42 °C for 600 s, the surface temperature of the garment reached 31.7 °C, indicating its potential application in wearable protective equipment.

Inspired by the structural features of the Parisian swallowtail butterfly wing (Figure 9C(i)), Li et al. [149] developed a multifunctional grid-based thermal energy converter (SETC) for economical storage of renewable solar and thermal energy (Figure 9C(ii)). The SETC demonstrated high solar absorption (~94%) and excellent electrical conductivity (6622 S cm^−1^). It could dynamically track the retreat of the solid-liquid phase interface.

Phase change materials (PCMs) enable rapid thermal response (<1 min) and high energy storage efficiency, with solar thermal storage efficiency of around 90.1% and electrical storage efficiency of approximately 86.1% (Figure 9C(iii)). This dynamic charging approach effectively mitigates long-distance heat transfer limitations and overcomes the low thermal conductivity of phase change materials and the thermal resistance between the charger and PCM. Phase change behavior is another critical mechanism; biomimetic structures stimulated by polar bear fur (Figure 9D(i)) have been transformed into composite phase change materials (CPCMs). Their “brick-and-mortar” microstructure absorbs and releases latent heat near the phase transition temperature, achieving spectral-selective thermal storage and maintaining temperature stability in extreme environments. Lin et al. [150] developed a wood-based phase change material (PWPCM), fabricated via in situ polyurethane polymerization within lignin-free wood (Figure 9D(ii)). PWPCM exhibited high strength, with a longitudinal tensile strength of 81.9 MPa, and excellent thermal energy storage capacity, with an enthalpy of 116.1 J g^−1^. Additionally, it demonstrated superior temperature regulation and shape stability, as illustrated in Figure 9D(iii).

#### 3.3.2. Photoinduced Self-Adaptation

**Figure 10:**
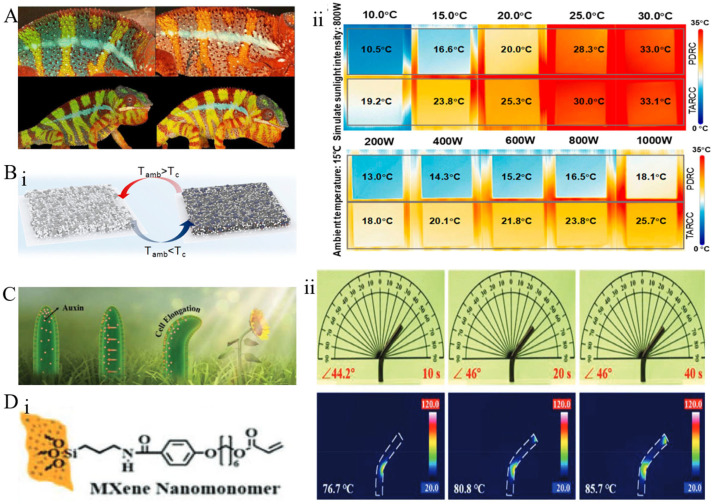
Biological photoadaptive responses: (**A**) Dynamic spectral modulation in chameleons [142]: Images illustrating color changes in two male chameleons induced by reversible dynamic spectral modulation. (**B**) (**i**) Schematic diagram of the TARCC structure [152]; (**ii**) comparative temperature analysis between TARCC and PDRC coatings. (**C**) Photoadaptive spectral modulation in sunflowers [157]: schematic illustration of phototropic mechanisms in plants, powered by auxin gradients and heliotropic growth towards sunlight. (**D**) (**i**) Structural schematic of MXene-LCE soft actuator tubes [157]; (**ii**) actual images of the elastic actuator during light tracking and corresponding thermal imaging photographs.

Solar cells, spacecraft, and similar systems require stable thermal regulation under fluctuating sunlight; however, existing materials struggle to dynamically balance light absorption and heat dissipation [151]. In contrast, biological systems such as chameleons exhibit rapid color changes through spectral modulation during camouflage. Notably, adult male panther chameleons (*Furcifer pardalis*) display additional intraspecific color variations [142]. When confronting rival males or preferred females, these chameleons can shift their background skin color from green to yellow or orange, with blue patches turning white, reds intensifying, and overall hue changes being less pronounced (Figure 10A). In stimulated skin, the spacing of guanine crystals within the iridophores is actively expanded, selectively reflecting red-shifted wavelengths, resulting in the transition of the skin from green to yellow/orange. Conversely, in red skin regions, numerous iridophore cells are hereby replaced by red pigmented granules, resulting in minimal hue variation during stimulation, with only increased brightness. Guided by this mechanism, researchers [152] developed a thermally adaptive radiative cooling coating (TARCC) that can dynamically modify its optical properties (Figure 10B(i)). This coating employs thermochromic microcapsules that switch between solar absorption and reflection based on temperature. During summer, TARCC appears white, reflecting 93% of solar radiation and emitting 94% of its thermal radiation (ATW), achieving cooling below ambient temperature by 6.5 K. In winter, it darkens, absorbing visible light and reflecting 50% of solar radiation, resulting in a temperature elevation of 4.3 K above ambient (Figure 10B(ii)).

Sunflowers [153], through their unique behaviors and structures (Figure 10C), achieve effective thermoregulation by utilizing differential auxin distribution within their stems to orient their inflorescences toward the sun, thereby maximizing light absorption efficiency. Beyond sunflowers, most plants in nature exhibit phototropism, growing toward light to optimize photosynthesis and enhance surface temperature to draw insect pollinators [154]. Organisms not only perceive the direction of sunlight but also respond by continuously monitoring the sun’s movement, demonstrating a form of self-regulating biological intelligence. This bending behavior results from deformation gradients along the stem’s diameter. In photoresponsive materials, light-induced contraction generates radial deformation gradients, causing movements analogous to plant bending toward sunlight [155]. Phototropism fundamentally involves a light-responsive process, with numerous Bio-inspired Transforming Materials (BITMs) designed based on the principles of phototropism. The most common approach involves BITMs composed of light-absorbing agents and thermosensitive polymers, where incident light is initially captured and transformed into heat by the light-absorbing component [156]. This heat then induces deformation within the thermosensitive polymer matrix. Feng et al. [157] reported a biomimetic phototropic MXene-enhanced liquid crystal elastomer tubular actuator with a hollow (Figure 10D(i)), radially symmetric structure capable of autonomous full-range lightweight tracking. The hollow architecture slows radial heat conduction, establishing a significant temperature gradient between illuminated and shaded surfaces, resulting in localized contraction and bending of the actuator toward the light source (Figure 10D(ii)). This system exhibits rapid photoreactivity and high-precision tracking. Such biomimetic designs, by dynamically adjusting the incident light angle, maximize light absorption while minimizing thermal energy loss, offering an optimized way for solar panels, concentrators, and similar devices.

#### 3.3.3. Humidity-Induced Adaptive Regulation

**Figure 11:**
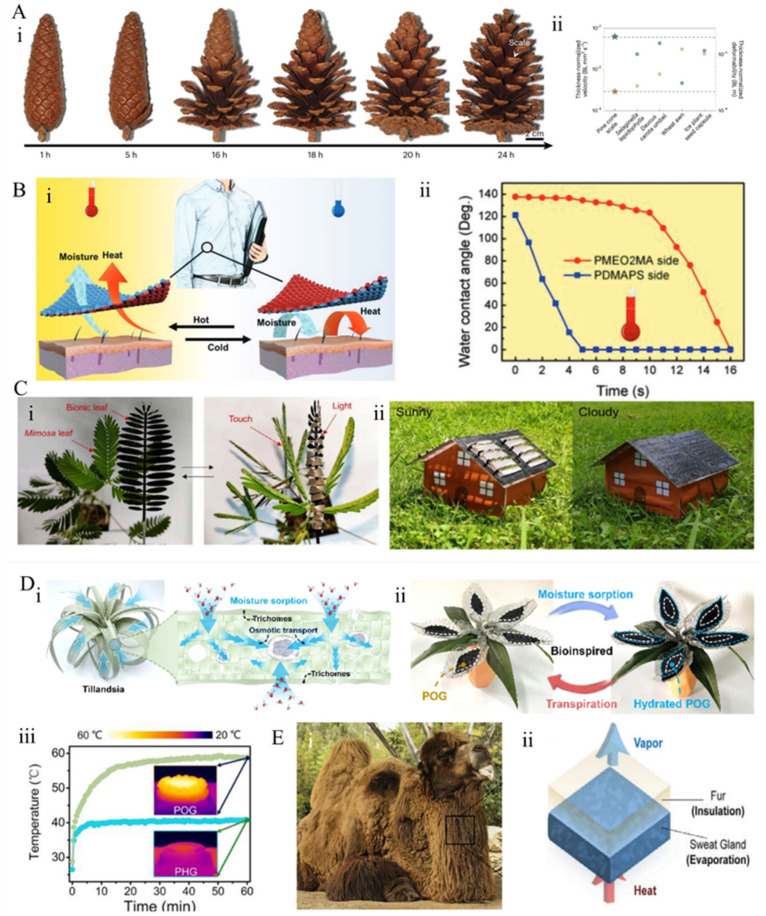
Biological humidity-induced self-adaptation: (**A**) Conifer scale [159] (**i**) the opening process of water-immersed cones. Over time, the water-immersed cones gradually open at 35 °C and 30–40% RH, requiring 24 h to reach full opening; (**ii**) a summary of deformation rates and capacities of various hygroscopic biological structures, with conifer scales (stellate) exhibiting the minimal normalized thickness change rate (brown symbols) while maintaining considerable deformation ability (green symbols). (**B**) (**i**) Schematic diagram illustrating the working mechanism of intelligent DB-Janus fabric in temperature-responsive humidity regulation and thermal management [162]; (**ii**) quantitative changes in water contact angle (WCA) at 40 °C. (**C**) Mimosa leaf and biomimetic leaf [164]: (**i**) unfolding without external stimuli (left) and folding upon touch/shaking (right); (**ii**) a house model covered with LAS under natural sunlight and shade (28 °C, 60% RH). (**D**) *Tillandsia*-inspired hygroscopic POG concept [165]: (**i**) schematic of *Tillandsia* absorbing moisture from the air for its physiological functions; (**ii**) illustration of the hygroscopic POG inspired by *Tillandsia*, capable of mimicking its water adsorption and transpiration behaviors; (**iii**) temperature-dependent changes of POG and Photothermal Hygroscopic Gel (PHG) over time. (**E**) (**i**) Photograph of a Bactrian camel [166]; (**ii**) layered skin design featuring sweat glands for evaporation-based cooling and porous fur providing insulation to mitigate environmental heat gain.

Humidity is a critical environmental factor in nature, and numerous organisms have evolved adaptive thermoregulatory mechanisms in response to these conditions. Plants exhibit unique adaptive movements in response to external stimuli, effectively managing their thermal energy [158]. For instance, pine cone scales [159] are composed of hygroscopic cellulose layers that swell and close at high humidity levels, thereby reducing seed exposure to external heat or rain. Conversely, at low humidity, they contract and open, facilitating seed dispersal and regulating internal ventilation for heat dissipation (Figure 11A(i,ii)). Inspired by biological processes, the development of humidity-responsive BITMs has garnered significant interest. A widely adopted strategy involves incorporating humidity-sensitive functional groups into polymer networks, where plasticization effects lower the polymer’s phase transition temperature, enabling shape recovery at lower or ambient temperatures [160,161]. Wang et al. [162] reported a temperature-responsive bilayer Janus fabric (Figure 11B(i)), with each side coated with polymers of different analytical dissolution temperatures. At elevated temperatures, hydrophobic methyl acrylate groups are exposed, promoting evaporative cooling; at lower temperatures, hydrophilic polyethylene glycol side chains are disclosed, providing insulation. This fabric maintains a surface temperature 1.2–2.3 °C lower than pure cotton at elevated temperatures and 3.3 °C higher at low temperatures, demonstrating superior thermal management performance (Figure 11B(ii)).

As humidity increases, hydrophilic groups within the material’s structure absorb moisture from the environment, leading to an expansion of molecular chain spacing and subsequent material swelling [163]. Conversely, when humidity decreases, desorption of water causes the material to become dehydrated. Inspired by the folding behavior of Mimosa leaves upon touch or agitation (Figure 11C(i)), Liu et al. [164] designed a Light-Adaptive Self-folding Sheet (LAS) that exploits asymmetric expansion between an active layer and a passive layer to achieve light-responsive self-folding motion in response to fluctuating sunlight. Under illumination, the active layer undergoes photothermal heating and dehydration, inducing negative thermal expansion, while the passive layer exhibits typical positive thermal expansion. This asymmetric thermal expansion causes the membrane to distort under light exposure. The membrane can achieve large bending angles within a relative humidity range of 10–90%, with response speed increasing as humidity rises. By modifying the membrane’s curvature and aperture, the area of the silver reflective layer can be modulated to facilitate temperature regulation. Indoor experiments showed that the LAS system maintained temperatures 27 °C and 4 °C higher than systems without LAS during cooling. In a three-day outdoor test, the LAS/BA system’s minimum temperature was 23 °C higher than that of the Base Assembly (BA) system (Figure 11C(ii)), both results indicating that LAS significantly enhances thermal management and solar heat storage efficiency.

Air plants (*Tillandsia*) [165] thrive without soil by directly absorbing water and nutrients from the atmosphere through their leaves (Figure 11D(i)). Their scale-like structures facilitate effective heat dissipation by maximizing surface area, thereby enhancing thermal loss efficiency. This unique combination of structural and functional characteristics enables air plants to maintain relatively stable internal temperatures across diverse ecological conditions. Inspired by this natural design, Ni et al. [165] developed a biomimetic hygroscopic photothermal organic gel (POG) (Figure 11D(ii)) for atmospheric water harvesting driven by solar energy. The POG exhibits an equilibrium moisture absorption capacity of 16.01 kg m^−2^ at 90% relative humidity and achieves a water production rate of up to 2.43 kg m^−2^ per day in outdoor experimental settings (Figure 11D(iii)). This material demonstrates multiple advantageous properties, including self-sustaining behavior, high water uptake, mechanical flexibility, and robust photothermal performance. Collectively, these functionalities enable efficient and continuous moisture absorption and thermal management across a wide range of humidity levels.

Inspired by mammalian sweating behavior, hydrogel-based evaporative cooling is regarded as an effective strategy for reducing energy consumption. Lu et al. [166] developed an evaporative insulation cooling system stimulated by the fur layer of desert animals such as camels, comprising a transparent bilayer structure of hydrogel and aerogel (Figure 11E(i)). Cooling is achieved through hydrogel evaporation, while the low thermal conductivity of the aerogel minimizes heat transfer from the environment (Figure 11E(ii)). Compared to single-layer systems, this bilayer design extends the cooling duration by approximately 400%, with a lower evaporation rate, resulting in a significantly prolonged cooling period. The high cooling efficiency, combined with prolonged cooling duration, plays a crucial role in optimizing thermal management.

#### 3.3.4. Mechanism-Induced Adaptive Response

**Figure 12:**
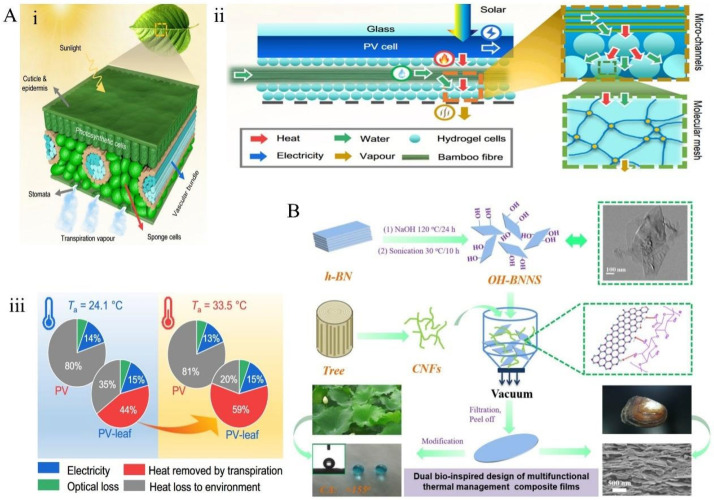
Biological mechanical stimuli induce adaptation: (**A**) (**i**) the typical internal structure of a real leaf [167]; (**ii**) the internal architecture of biomimetic transpiration structures; (**iii**) energy distribution analysis under varying environmental temperatures. (**B**) Schematic diagram of the fabrication process for OH-BNNS and CNFs/OH-BNNS composite films [168].

Some organisms have evolved sophisticated mechanisms capable of sensing and reacting to external mechanical stimuli—such as contact, pressure, or deformation—thereby triggering dynamic restructuring of their surface or internal structures. This mechanically induced adaptive behavior enables biological systems to modulate their optical, thermal, or fluidic properties in real time, optimizing functions such as camouflage, thermal regulation, or energy harvesting. Mechanically adaptive materials achieve intelligent thermal management through structural deformation or the optimization of heat flow pathways via pre-designed microstructures, without requiring external energy input. Inspired by natural microfluidic systems in leaf venation [167] and the “brick-and-mortar” architecture of nacre [168], these materials utilize mechanical forces or structural reorganization to dynamically regulate thermal conductivity and distribution. The transpiration mechanism of leaf venation [167] (Figure 12A(i)) has inspired the PV-leaf system (Figure 12A(ii)), which employs bamboo fiber bundles and hydrogel units to emulate plant transpiration, effectively removing approximately 590 W/m^2^ of heat during photovoltaic thermal management. This narrows the temperature by 26 °C under 1000 W/m^2^ irradiation and enhances solar energy utilization efficiency from 13.2% to 74.5% (Figure 12A(iii)). This biomimetic microfluidic design achieves high-efficiency cooling through passive water evaporation, eliminating the need for external pumps or control systems, thereby exemplifying structure-driven thermal self-adaptation.

On the other hand, the highly ordered lamellar structure of the nacreous layer [168] offers a new paradigm for thermal conductivity optimization. In its “brick-and-mortar” model, the rigid BN platelet layers [169] are oriented through 3D printing supported by rotary scrapers, forming biomimetic layered architectures. This biomimetic design significantly reduces interfacial thermal resistance, elevating the thermal conductivity from 0.29 W·m^−1^·K^−1^ to 7.69 W·m^−1^·K^−1^ (a 25.5-fold increase), and exhibiting a 5.8-fold anisotropy in thermal conduction (Figure 12B). The tight interlayer bonding and staggered stacking of BN effectively direct heat flow, confirming that mechanical structural design plays a critical role in thermal management performance. Such materials demonstrate considerable potential in electronic heat dissipation applications.

Table 2 clearly shows that biomimetic strategies have led to multiple high-performance cooling designs, but there are often trade-offs between scalability, cost, and durability. For example, photonic crystal-based designs have excellent performance but high cost; Designs based on biopolymers (wood, silk) or coatings are easier to scale, but their long-term environmental durability still needs to be further verified. This comparison provides a clear guide for researchers to choose the appropriate technology path for specific application scenarios, such as large-scale architectural coatings, high-end electronic cooling, or wearable textiles.

## 4. Practical Biomimetic Radiative Cooling Technology

Unlike conventional mechanical refrigeration, passive radiative cooling relies on the optical properties of materials to achieve cooling effects without any energy consumption. This approach offers opportunities to mitigate energy crises and accelerate global decarbonization efforts. Although the cooling capacity of PDRC devices currently does not respond to meeting large-scale energy-intensive demands, they can serve as supplementary systems to traditional cooling methods, thereby reducing overall cooling energy consumption. PDRC technology can be directly incorporated into buildings, personal thermal management systems, or other devices. This section will examine the practical applications of PDRC in energy-efficient architecture, individual thermal regulation, electronic device cooling, and water harvesting.

### 4.1. Building Energy Efficiency

Buildings are the most important consumers of energy, accounting for approximately 39% of total energy demand, thereby significantly exacerbating the global energy crisis [170]. During hot summer months, cooling systems such as air conditioning are typically used to reduce indoor temperatures, which not only entails substantial energy consumption but also intensifies greenhouse gas emissions [171,172]. The deployment of energy-efficient PDRC devices offers a sustainable solution for building cooling, markedly reducing energy use. Under favorable climatic conditions, PDRC technology can provide up to 80% of the net zero cooling load for buildings [173,174]. The integration of radiative cooling materials with building envelope components effectively mitigates the cooling load within indoor spaces, issuing an energy-efficient, environmentally friendly, and high-performance cooling strategy.

**Figure 13:**
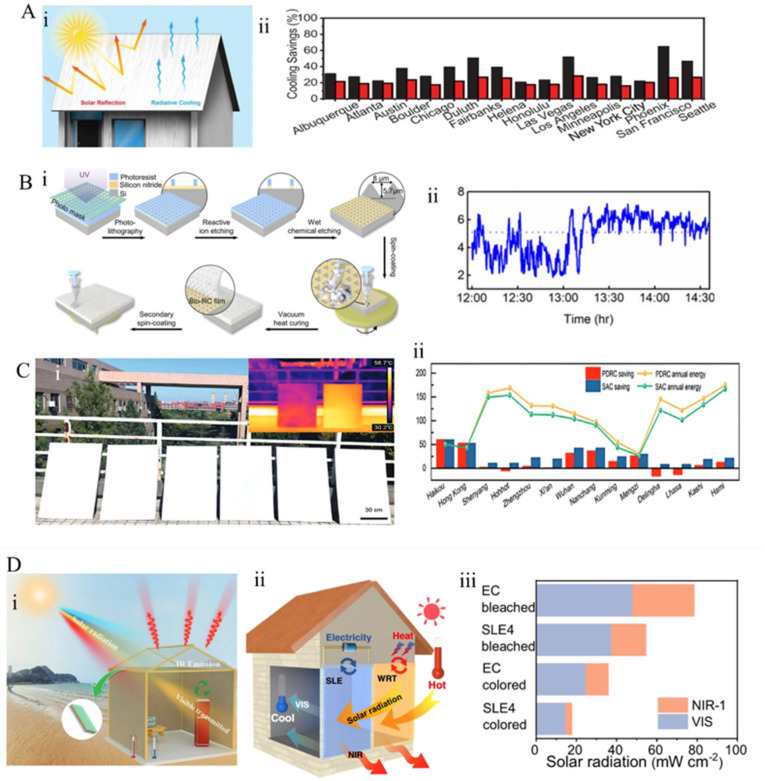
Radiative cooling applications in building energy efficiency: (**A**) (**i**) cooling timber for architectural cooling [76]; (**ii**) cooling losses in buildings in the southern United States. (**B**) (**i**) Fabrication of Bio-RC thin films [75]; (**ii**) achieved cooling effects. (**C**) (**i**) Large-scale fabrication of SAC coatings [179]; (**ii**) energy consumption modeling. (**D**) (**i**) Mechanisms of window regulation [181]; (**ii**) manufacturing of intelligent windows; (**iii**) radiative performance of smart windows.

As the surface of the roof is directly exposed to solar radiation, it is essential to take full advantage of the synergistic effects of solar reflectance and infrared emissivity. For instance, Li et al. [76] investigated the potential energy-saving benefits of cooling wood with high solar reflectance and infrared emissivity as a building envelope material (Figure 13A(i)). Computational results for mid-rise apartment models suggest that cooling-treated wood can reduce cooling energy consumption by 20–35%, with this benefit extending to all U.S. cities, particularly in the southern regions (Figure 13A(ii)). Applying radiative cooling coatings directly onto external roof surfaces presents a more attractive option, as it can greatly reduce building cooling loads and energy consumption [175,176,177,178]. In this context, Zhou Han’s team at Shanghai Jiao Tong University [75] replicated the micro-pyramid structure of beetle elytra via photolithography, fabricated silicon templates, and spin-coated precursor solutions containing organosilicon and alumina microspheres. Post-thermal polymerization, they obtained flexible biomimetic films (Figure 13B(i)). When applied to roof building, these coatings achieved a surface temperature reduction of 5.1 °C under an incident solar intensity of 862 W/m^2^, with the black substrate surface cooling by over 18 °C (Figure 13B(ii)). To address the issue of increased heating loads during winter associated with traditional radiative cooling roofs, Nanjing University of Technology [179] proposed a ventilated radiative cooling roof inspired by the multilayer insulation mechanism of polar bear fur (Figure 13C(i)). By integrating biomimetic coatings with ventilated cavities, this design reduced summer cooling energy consumption by 21.8%, decreased winter heat loss by 16.9%, and greatly improved overall annual energy savings (Figure 13C(ii)). Further innovation stems from thermochromic roof coatings inspired by chameleon skin’s temperature-dependent infrared transmittance. Dang et al. [180], utilizing ion gel gating induced by triboelectric nanogenerators (TENGs), modulated hydrogen ion insertion/extraction within VO_2_ crystal lattices at room temperature, inducing a phase change that controlled optical transmittance. This phase transition caused a sharp 28.1% decrease in VO_2_’s infrared transmittance, thereby enhancing insulation performance. Additionally, a thermosensitive powder coating based on VO_2_ phase-change material developed by a team in Ningbo [181] maintained high reflectance (0.71) at low temperatures and transitioned to a high-emissivity state at elevated temperatures.

Finally, and equally important, for radiative cooling materials used in building applications, in addition to the fundamental requirement of cooling performance, other properties must be evaluated. These include mechanical strength [182,183], anti-fouling self-cleaning capabilities [176,184], weather resistance [15,185], adhesion to building surfaces [186], scalable fabrication processes [111], environmental sustainability [76,187], aesthetic appeal [188,189], and cost considerations [190,191].

Windows are an essential medium for architectural lighting and a significant factor in thermal energy loss within buildings (Figure 13D(i)) [192]. Particularly during summer, the greenhouse effect caused by traditional glass windows presents a diagnostic challenge, substantially increasing indoor cooling loads and energy consumption. Inspired by the extraordinary photon management structure of the Hercules beetle’s exoskeleton, Zhang et al. [182] developed a tri-layer smart glass (Figure 13D(ii)), featuring a top DMD (dielectric/metal/dielectric) multilayer film that reflects approximately 88% of near-infrared heat, while the mid-layer glass substrate maintains a visible light transmittance of 63% (Figure 13D(iii)). The bottom PDMS radiative layer exhibits an emissivity of up to 95%.

### 4.2. Personal Thermal Management

**Figure 14:**
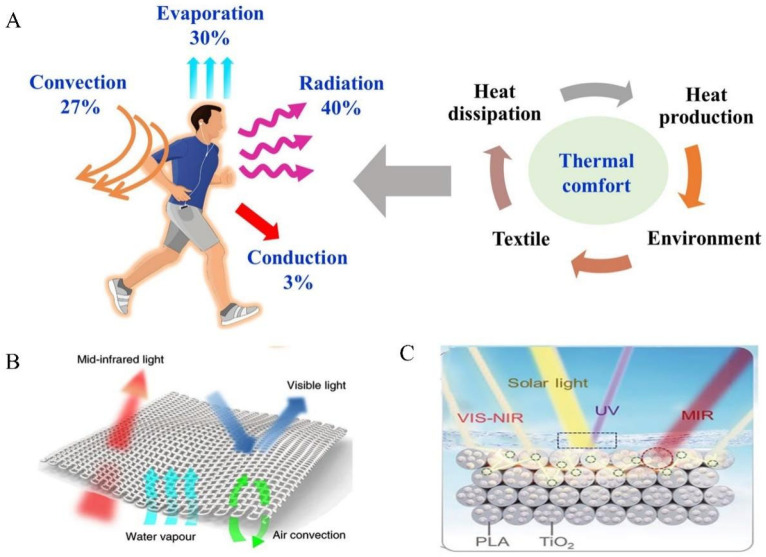
Personal thermal management: (**A**) the pathways of heat dissipation from the human body and four key factors influencing thermal comfort [193]. (**B**) Nanoporous polyethylene ultrafine fibers [198], exhibiting high mid-infrared transparency, visible light shielding, and excellent wear resistance. (**C**) Schematic diagram of a *metafabric* with a PDRC three-layer hierarchical structure [201].

The human body continuously generates metabolic heat, which is shared with the surrounding environment through radiation, conduction, convection, and evaporation to maintain thermal equilibrium [193]. Thermal comfort is a critical indicator of this balance and is closely connected with heat transfer between the skin, textiles, and the environment (Figure 14A). As an interface between the human body and its environment, textiles play a critical role in mediating heat exchange. Common textile materials exhibit infrared absorption wavelengths overlapping with atmospheric windows, resulting in strong absorption of radiated heat from the human body [194]. Consequently, various strategies have been suggested to develop radiative cooling textiles aimed at achieving personal thermal regulation. Built on the cooling mechanism, radiative cooling textiles can be categorized into mid-infrared transparent textiles and mid-infrared emissive textiles.

The skin acts as a thermal radiator capable of radiating heat through mid-infrared transparent materials. Polymers such as polyethylene, polyamide, polyester, and polycaprolactam exhibit high mid-infrared [195,196] transparency due to limited vibrational modes within the MIR range. When fiber diameters are similar to visible light wavelengths, strong Mie scattering maintains visible opacity, making these fibers suitable for mid-infrared transparent cooling textiles [197]. For example, Cui et al. [198] mimicked the hierarchical porous structure of silkworm cocoon fibers by producing uniform, continuous nanostructured porous polyethylene fibers via melt extrusion and paraffin oil as a pore template. These ultrafine fibers, which possess cotton-like softness, demonstrate excellent cooling performance and are suitable for industrial textile production (Figure 14B).

The mid-infrared emissivity of such textiles is given in the vibrational modes of molecular bonds and functional groups [199,200]. For instance, silk fibers inherently exhibit high emissivity, and layered structures that enhance solar reflectance further improve their suitability for cooling textiles [134]. Recently, PDRC has reported multilayer superfabric composites comprising TiO_2_-poly(lactic acid) woven fabrics laminated with thin PTFE layers, which mimic the core–shell structure of hollow fibers found on the underside of poplar leaves [201] (Figure 14C). Through layered design and random scattering, these superfabric structures achieve high solar reflectance and elevated mid-infrared emissivity. The development of PDRC textiles offers a promising approach for cooling localized microenvironments of the human body and enhancing personal thermal comfort. However, scalable manufacturing processes must also consider wearable console attributes such as flexibility, breathability, and moisture permeability.

### 4.3. Electronic Devices

In recent years, cooling electronic chips and flexible electronic devices using PDRC materials have garnered significant attention. As technological advancements continue, the miniaturization of electronic components has intensified, exacerbating thermal management challenges [202,203]. Over 55% of microelectronic device failures are assigned to elevated operating temperatures [204], underscoring the critical importance of effective cooling strategies to enhance device stability and longevity. Central to these approaches is biomimicry of optical and thermal regulation mechanisms, leading to the development of intelligent materials that combine high solar reflectance with high infrared emissivity to address the severe heat dissipation issues associated with device miniaturization and high power densities.

**Figure 15:**
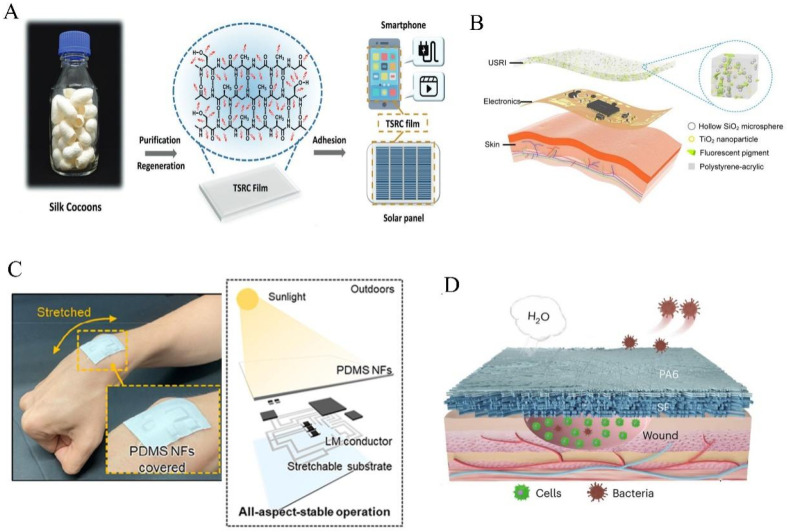
Radiative cooling applications in electronic devices: (**A**) schematic illustration of the transformation of opaque cocoons into TSRC thin films [206] and their application in radiative cooling of electronic equipment; (**B**) components and assembly methods for ultrathin flexible radiative cooling interfaces [207]; (**C**) outdoor wearable electronic devices composed of polydimethylsiloxane nanofibers and liquid metal conductors [208] (**D**) schematic design of PDRC dressings for localized wound environment management under sunlight [209].

Radiative cooling technology demonstrates substantial potential for passive, energy-free thermal management of electronic systems, with its application scope continually expanding [205]. Initially, radiative cooling films fabricated from naturally transparent silk materials attracted interest due to their optical properties. These materials emulate the natural transparency and high infrared emissivity of silkworm cocoon proteins, enabling nonstop coverage of light-sensitive regions such as electronic displays to facilitate passive heat dissipation—though this approach is limited to specific scenarios (Figure 15A). More broadly, high solar reflectance PDRC devices are employed to cool various electronic devices, utilizing base materials such as silver nanowire coatings on multilayer porous films. These not only meet cooling requirements but also enable the fabrication of flexible electronics with electrical performance comparable to traditional rigid hardware [206].

To further enhance the thermal management capabilities and conformability of flexible electronics, ultrathin PDRC structures incorporating functional materials within polymer matrices have been developed. Addressing issues of breathability and thermal stability, one solution involves electrospinning thermosetting polymer nanofiber membranes (Figure 15B) [207]. These membranes overcome comfort limitations through their excellent structural permeability while maintaining superior radiative cooling performance owing to high solar reflectance and mid-infrared emissivity (Figure 15C). Such nanofiber membranes can even be integrated with liquid metal conductors to construct safe, stable wearable systems. However, it is important to note that polymer nanofiber membranes typically exhibit limited mechanical strength, especially under extreme environmental stresses, which may compromise durability. Therefore, rigorous evaluation and assurance of their robustness are critical to practical applications and technological maturation.

Beyond flexible electronics, nanofiber membranes with radiative cooling properties have demonstrated potential in wound healing applications. Inspired by the porous chitinous network of butterfly wing scales, layered dressings composed of polyamide and silk fibroin leverage Polyamide 6(PA6)’s high infrared transmittance and SF’s biocompatibility and high infrared emissivity to achieve dual functions of passive cooling and biological repair. Optically, these dressings reflect up to 96% of sunlight and emit 94% in the mid-infrared range, radiating heat through the atmospheric transparency window (8–13 μm) into outer space, thereby reducing wound surface temperature by approximately 7 °C under direct sunlight [208] (Figure 15D). Biologically, the micro/nanofiber network imparts excellent breathability, moisture permeability, and antibacterial properties, effectively blocking pathogenic invasion such as Staphylococcus aureus while holding a moist wound environment. Compared with conventional dressings, this innovative material significantly suppresses inflammation caused by elevated local temperatures in outdoor hot environments. Animal studies reveal that wounds covered with this dressing maintain temperatures below 36 °C, whereas control groups exceed 37 °C. RNA sequencing confirms downregulation of inflammation-related genes and upregulation of skin regeneration genes, with modulation of chemokine signaling pathways accelerating collagen deposition and re-epithelialization, ultimately enhancing wound healing rates. This technological breakthrough offers a zero-energy solution for wound management in risky environments and provides insights into the molecular mechanisms of thermal microenvironment regulation in tissue repair.

### 4.4. Thermoelectric Generator

**Figure 16:**
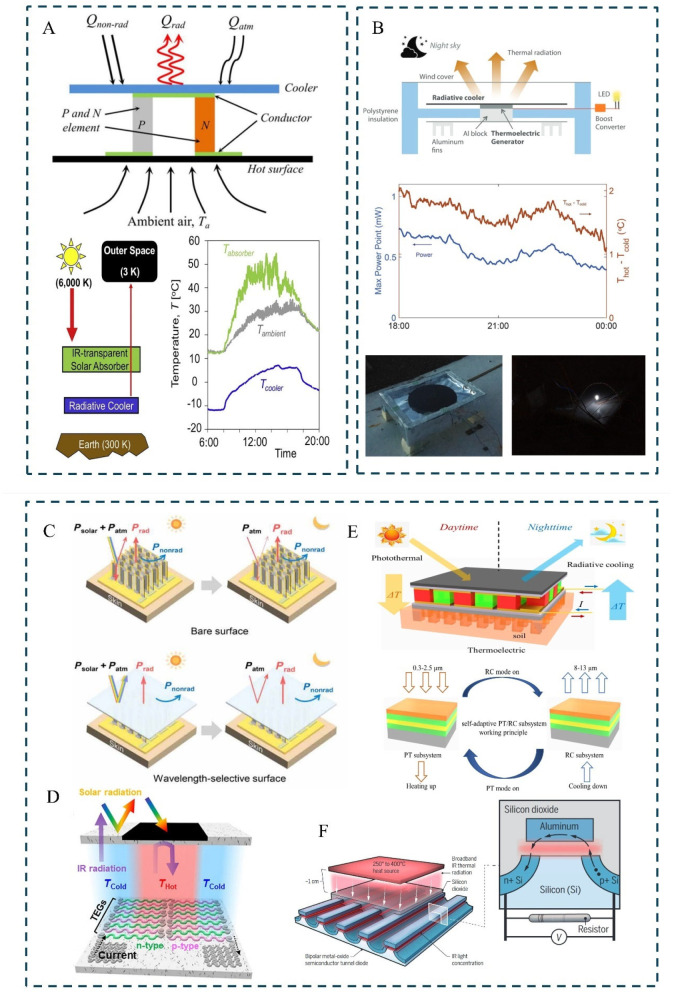
Schematic illustration of radiative cooling applications in thermoelectric power generation (**A**) and integration of TEG devices with PDRC cells [212]; (**B**) schematic of a low-cost nocturnal radiative cooling thermoelectric generator [213]; (**C**) wearable thermoelectric generator: heat transfer process with wavelength-selective surface TEGs [215]; (**D**) an energy harvesting system comprising PEDOT:PSS-patterned PLCL microfiber membranes serving as radiative cooling/heating components for the full-day thermal gradient, along with n- and p-doped silicon arrays for TEGs [214] (**E**) operational principle of an adaptive PT/RC-TE system and PT/RC sub-system, enabling mode switching between PT and RC modes [216]; (**F**) bipolar semiconductor tunnel diode capable of converting infrared (IR) radiation from low-temperature heat sources into electrical energy [217].

Although photovoltaic technology has addressed daytime power supply issues, the challenge of sustainable nighttime electricity generation remains a technological bottleneck. A synergistic system combining radiative cooling and thermoelectric generators (TEGs), which catches the temperature differential between the cold space radiative source and the environmental heat source, offers an innovative pathway for power supply under non-illuminated conditions [209,210]. Integrating PDRC devices onto the surface of TEGs enables power generation even during nighttime. The core principle mimics the heliotropic behavior of sunflowers, dynamically switching between photothermal and radiative cooling modes. The PDRC device acts as an active remote side for the TEG, continuously radiating heat into the cold outer space, while the hot side naturally absorbs ambient heat or is heated by other thermal sources, establishing a significant passive temperature gradient. This temperature differential is the fundamental driving force for thermoelectric conversion (Figure 16A) [211]. Research indicates that optimized designs—such as coupling radiative coolers with high-emissivity near-blackbody surfaces—not only facilitate effective radiative heat dissipation at night, driving TEG power generation and directly illuminating low-power devices like Light-Emitting Diodes (LEDs) [212] (Figure 16B), but also provide potential passive power solutions for wearable electronics and Internet of Things (IoT) nodes. In this context, human skin serves as a convenient thermal source for the hot side, while the surrounding environment contributes to the unsympathetic side effect [213].

To overcome the limitations of traditional structures, an innovative approach involves arranging the cold (PDRC region) and hot ends in alternating, strip-like parallel configurations. This design effectively addresses the constraints of unidirectional heat transfer in terms of shape adaptability and large-scale scalability, enabling efficient generation of temperature gradients over extensive surfaces to drive TEG power generation [214]. However, challenges persist; most photon management-based TEG systems currently work on static, single-mode conditions.

To achieve all-weather energy harvesting (Figure 16C), research has shifted toward developing adaptive systems [215]. Such systems can intelligently switch operational modes in response to diurnal and nocturnal environmental changes (Figure 16D), activating photothermal mode during the day to maximize solar absorption and suppress radiative heat loss, thereby ensuring effective heating of the hot side; and seamlessly transitioning to radiative cooling mode at night, optimizing heat dissipation at the TEG’s top to maintain the low temperature of the cold side (Figure 16E) [216]. This dynamic regulation of spectral absorption/emission essentially mimics biological responses to environmental variations, aiming to efficiently collect and utilize two major renewable thermal energy sources: solar and deep space cold energy (Figure 16F).

In summary, integrating biomimetic radiative cooling technology into thermoelectric generator design significantly enhances device performance, particularly in the recovery of ecological and human residual heat and in wearable power applications. This advancement lays a crucial foundation for the development of next-generation high-performance TEGs. Nonetheless, effectively overcoming the intrinsic limitations of energy density and power density in TEGs while fully leveraging the advantages of PDRC remains a diagnostic challenge and a central focus for future research to translate this integrated technology into practical applications.

### 4.5. Water Collection

**Figure 17:**
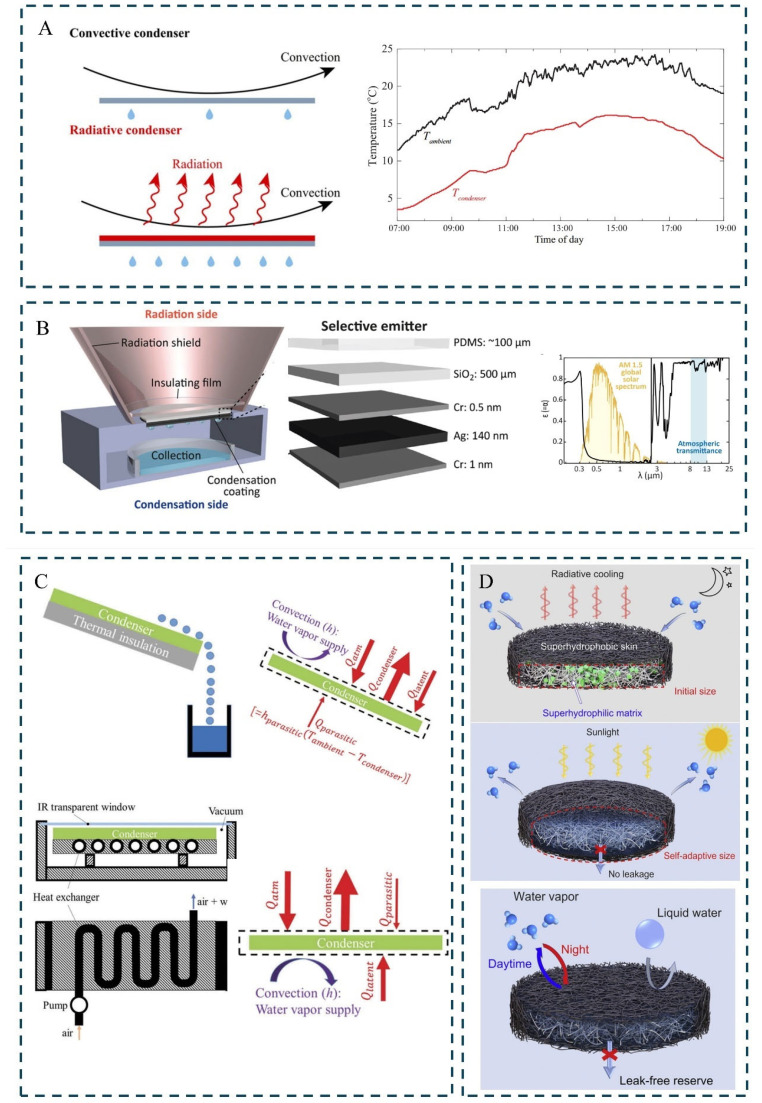
A schematic diagram of water collection applications utilizing radiative cooling: (**A**) convection condenser and radiative condenser [221]; (**B**) operational principles of separated radiative and condensing sides [222]; (**C**) integration of solar-driven interface seawater desalination with passive radiative cooling for continuous freshwater harvesting [225]; (**D**) schematic of a biomimetic heterogeneous wettability atmospheric water harvesting device [224].

Due to climate change, population growth, and water pollution, water scarcity is becoming a global threat and challenge, particularly in many underdeveloped and arid regions [217,218]. PDRC devices can effectively reduce the surface temperature of objects. When the surface temperature of a PDRC device drops below the dew point, dew formation occurs, enabling water collection and demonstrating significant water harvesting potential [219]. Compared with traditional water collection systems relying on active cooling, PDRC devices enhance condensation efficiency through synergistic regulation of radiative and convective heat dissipation. This is achieved by biomimetic surface designs that attain 92.9% high reflectivity in the solar spectrum (0.3–2.5 μm) to minimize heat input, and 98.1% high emissivity within the atmospheric window (8–13 μm), passively radiating heat into the cold universe. Consequently, in environments with non-saturated humidity, a temperature reduction of approximately 6.9 °C below the dew point is achieved, directly inducing water vapor condensation [220] (Figure 17A). To further optimize this process, biomimetic structural designs are incorporated at a deeper level. For instance, constructing hydrophilic/hydrophobic heterogeneous microregions (contact angle 153°, roll-off angle 42°) can simultaneously optimize nucleation, coalescence, and droplet shedding behaviors. The introduction of shielding layer structures can also direct thermal radiation and block low-altitude atmospheric radiative interference, increasing condensation efficiency by over 30% as opposed to commercial nighttime water harvesters [221]. A representative example is the biomimetic water-harvesting material developed by the Huazhong University of Science and Technology team [222], which mimics the humidity-driven opening and closing mechanism of pine cone scales. Combining micro-extrusion compression molding, it forms a porous ellipsoidal structure. Outdoor testing shows a nightly water yield of up to 1244 g/m^2^, sufficient to meet an adult’s daily drinking water needs with just 2 m^2^ of deployment (Figure 17B).

However, the water collection capacity of single-mode PDRC remains constrained by environmental variables; daytime solar radiation diminishes cooling effectiveness, and low humidity in waterless regions directly limits vapor flux. To address this, adaptive systems have been developed, integrating dual-mode operation of photothermal and radiative cooling. During the day, solar energy drives adsorption–desorption cycles, while at night, the system switches to radiative cooling to accelerate condensation, enabling continuous water production [223]. Additionally, inspired by the layered sweat gland and fur structures of camels, encapsulating superhydrophilic adsorbents within hydrophobic elastic nanofiber networks can extend operational windows in arid environments. The network’s inherent radiative cooling capability further prolongs the high-efficiency working period of the adsorbent [224].

Although under optimized conditions (such as high humidity at night), outdoor measured water yield can reach 1244 g/m^2^ (approximately 1.244 L/m^2^), the current system’s average water yield in actual variable environments (approximately 0.62–1.24 L/m^2^/day) still falls far below the theoretical limit (approximately 1.8–2.2 L/m^2^/day) [226]. The performance limitations need to be analyzed from two aspects: the impact of the geometric structure of the radiative condenser, where cloud cover can obstruct the atmospheric window radiation channel, and the effect of excessively high wind speeds, which can exacerbate convective heat exchange, weakening the radiative cooling effect. Additionally, the influence of the dynamic characteristics of the adsorbent, such as adsorption/desorption rates, humidity response lags, and material regeneration capacity, limits the efficiency of water vapor capture and release (Figure 17C). In summary, radiative cooling technology offers a promising pathway for sustainable, cost-effective, and scalable atmospheric water harvesting, acting as a supplementary water resource. Furthermore, atmospheric water collection devices based on radiative cooling possess potential for integration with seawater desalination (Figure 17D) and other systems, enabling temporal operational flexibility.

## 5. Summary and Future Perspectives

Bio-inspired Radiative Cooling (BIRC), as an emerging strategy to deal with the high energy consumption and environmental pressures associated with conventional refrigeration, draws inspiration from the intricate thermal radiation regulation mechanisms of biological organisms. This approach offers innovative pathways for the development of highly efficient passive cooling technologies. Research has progressed from fundamental principle validation and performance enhancement to tackling practical application bottlenecks. An in-depth understanding of biological models continually pushes the performance limits of traditional passive radiative cooling materials. BIRC technology demonstrates broad application potential across diverse fields, including building envelope systems, personal thermal management textiles and apparel, electronic device heat dissipation, food preservation, water harvesting, and thermoelectric power generation. However, transitioning from laboratory experiments to large-scale deployment presents significant challenges: the precise replication of complex biological structures and the gap between low-cost, scalable manufacturing processes; the high dependency on specific biological models, which may limit universality and adaptability; and the need for comprehensive validation of long-term stability, durability, and performance under multi-physical field coupling conditions. These systemic challenges constitute critical barriers to the commercialization and energy-saving, carbon-reducing potential of BIRC technology.

Advancing BIRC toward widespread application hinges on developing cost-effective, environmentally friendly, and scalable manufacturing strategies. Current methods tailored to specific biological models often face limitations such as laboratory confinement, reliance on specialized biological materials, or solvent pollution risks, hindering industrialization. Future priorities include exploring more universal bio-inspired extraction pathways and establishing scalable, economically viable, and ecologically sustainable fabrication protocols. In this context, abundant and renewable natural biological materials offer notable advantages due to their low cost, biocompatibility, and biodegradability. Additionally, cutting-edge scalable manufacturing technologies must deeply integrate the complexity of bionic designs. Recovering from the limitations of traditional trial-and-error approaches and single-physical-field simulations, the integration of artificial intelligence (AI) presents a revolutionary breakthrough [225,227]. AI can rapidly decouple the complex relationships between biological radiation regulation mechanisms and structural features, enabling intelligent inverse design of optimized structures adapted to specific applications. AI-based multi-level modeling and simulation platforms facilitate precise spectral property prediction and adaptive optimization, as well as long-term evolution simulations of structures under real-world ecological stresses. Furthermore, AI-driven intelligent process control enhances manufacturing precision and efficiency, reduces costs associated with complex structures, and ensures performance and durability, thereby bridging the entire chain from organizational design and material synthesis to device fabrication and performance evaluation. Future research should be focused on simplifying high-efficiency bionic structural design rules, strengthening environmental robustness assessments, and exploring the integration of diverse application scenarios.

BIRC mimics natural strategies by engineering materials with specific microstructures and spectral properties to achieve zero-energy cooling. Its core mechanism involves high emissivity in the atmospheric window (8–13 μm) for efficient heat radiation to space and high solar reflectance (0.3–2.5 μm) to minimize solar heat gain [228]. Current research focuses on developing tunable smart biomimetic materials integrated into building systems, such as combining radiative coolers with phase change materials for continuous day–night cooling or optimizing their integration with building envelopes [229,230]. The goal is to significantly reduce building cooling energy consumption, advance near-zero energy buildings, and provide an effective zero-carbon cooling solution to address global warming [231]. The field requires ongoing international research efforts to overcome challenges and accelerate the development of high-performance, adaptive, environmentally friendly radiative cooling devices, offering innovative multifunctional approaches to global cooling energy issues.

## Figures and Tables

**Table 1:** Basic materials for radiation cooling (advantages and disadvantages).

Material	Advantage	Disadvantage	Reference
Silicon	Good durability and stability	Sacrifice visible light transmittance	[29,30]
ZnS	Excellent chemical inertness	No reflective properties	[31,32,33]
polyphony oxide	Maintain humidity	Hard to process and mold.	[34]
Silicon Oxynitride	Modify chemical composition	Limited thermal stability	[35,36]
CdS	High IR band transmittance	Toxic	[37]
Al paint	High mechanical strength	Complex to make	[38,39]
Acrylic paint	Good adhesion	Less hard	[40,41]
TiO_2_ paint	No pollution	Coarser texture	[14,42]
Al_2_O_3_ paint	Prevent corrosion	Complex construction	[43,44]
TiO_2_ composite coatings	High solar reflectance	Unstable in high temperatures	[45,46]
Al_2_O_3_ composite coatings	Improves adhesion	Poor water resistance	[47]
Nano SiO_2_	High emissivity	Complex compounding process	[48,49]
Nano Al_2_O_3_	High mechanical properties	Complex purification process	[50,51,52]
Nano Polyacrylate	Good adhesion to the substrate	Insufficient weather resistance	[53,54]
Nanofibers	High surface area	Lack of durability	[55,56,57,58]
Nanocomposites	High reflectivity and emissivity	Complex modification process	[55,59,60,61,62]

**Table 2:** Comparison table of bionic radiant cooler performance and characteristics.

Biological Inspiration	Material	Optical Properties	Cooling Performance
Saharan Silver Ant	PDMS film	ε~MIR~: 0.98	High radiative power
Neocerambyx gigas	Micro-pyramid array polymer	R_solar_~: ~95% ε~MIR~: **>0.96**	ΔT: ~5.1 °C P_cool_: ~90.8 W/m^2^
Cyphochilus Beetle	Porous polymer films with micropyramidal structure	R_solar_~: ~98% ε~MIR~: **~96%**	High cooling power
Cicada Wing	Porous polymer–ceramic composite micro-spike structure	R_solar_~: **97.6%** ε~MIR~: **95.5%**	ΔT: **6.6 °C** P_cool_: **78 W/m^2^**
Poplar Leaf Trichomes	Hollow core–shell structure fiber membrane	R_vis_~: **60–70%**	Effective scattering and cooling
Koi Fish Scale	Multilayer photonic crystals	R_olar_~: **>97%** ε~ATW~: **High**	ΔT: **4.9 °C**
Wood	Delignin densifies wood	R_solar_~: **96%**	Building Energy Saving: **20–35%**
Silk	Electrospun silk fibroin membrane	R_solar_~: 95%	ΔT: **3.5 °C**
Thermochromic Chameleon Skin	Temperature-adaptive radiative cooling coating	Summer: R_solar_::93%, ε~ATW~: 94% Winter: R_solar_: ~50%	Summer: ΔT: −6.5 K Winter: ΔT: +4.3 K

## Data Availability

No new data were created or analyzed in this study. Data sharing is not applicable to this article.
